# Supplementary material for: Adaptable P body physical states differentially regulate bicoid mRNA storage during early Drosophila development
Source: Dev Cell. 2021 Oct 25;56(20):2886–2901.e6. doi: 10.1016/j.devcel.2021.09.021 (PMC8555633; doi:10.1016/j.devcel.2021.09.021)
Supplement: Document S1. Figures S1–S5 and Tables S1 [file mmc1.pdf]

Developmental Cell, Volume *56*

Supplemental information

Adaptable P body physical states differentially regulate *bicoid* mRNA storage during early *Drosophila* development

M. Sankaranarayanan, Ryan J. Emenecker, Elise L. Wilby, Marcus Jahnel, Irmela R.E.A. Trussina, Matt Wayland, Simon Alberti, Alex S. Holehouse, and Timothy T. Weil

Supplemental Information

Adaptable P body physical states differentially regulate *bicoid* mRNA storage during early *Drosophila* development

M.Sankaranarayanan, Ryan J. Emenecker, Elise L. Wilby, Marcus Jahnel, Irmela R. E. A. Trussina, Matt Wayland, Simon Alberti, Alex S. Holehouse, Timothy T. Weil

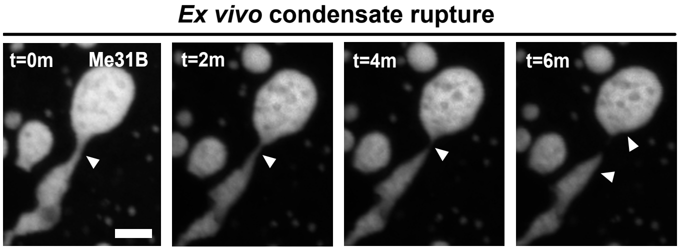

Figure S1. Extruded P bodies display condensate-rupture behavior. Related to Figure 2.

Extruded cytoplasm from a mature oocyte expressing Me31B::GFP. P bodies which have already fused are held together by a ‘bridge’ (t = 0 min, n = 10). Image sequence displaying ‘pinching off’ of the unstable bridge (white arrowheads point to the region of rupture). The two condensates resorb within a minute of bridge rupturing.

Scale bar: 2 μm.

A

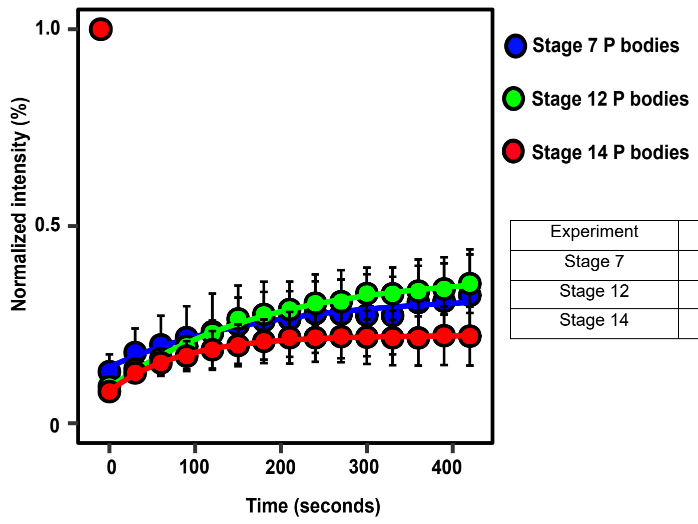

Experiment	Tau (seconds)	Mobile fraction	Immobile fraction
Stage 7	178.82	0.21	0.78
Stage 12	178.50	0.31	0.68
Stage 14	83.91	0.15	0.84

B

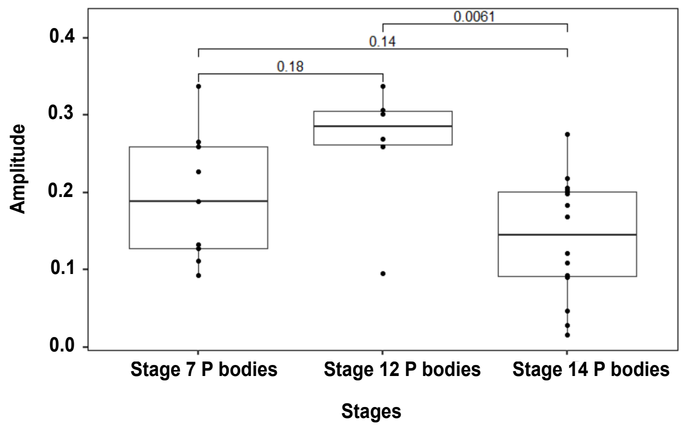

C

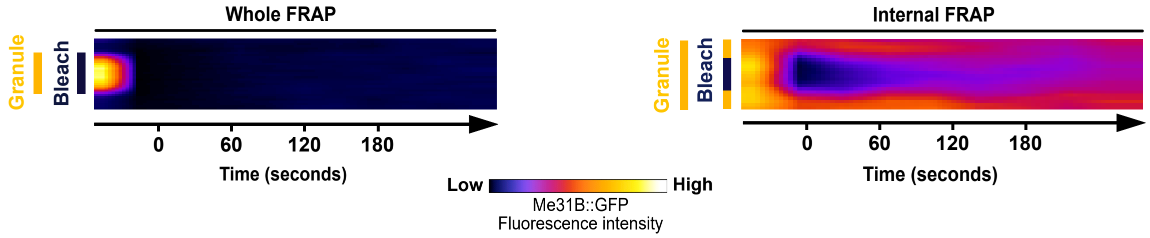

D

Experiment	Tau (seconds)	Mobile fraction	Immobile fraction
Whole FRAP	94.86	0.087	0.913
Internal FRAP	144.12	0.714	0.285

Figure S2. Me31B is mobile within P bodies but does not exchange with the cytoplasm. Related to Figure 2.

(A) Recovery profiles of whole FRAP of P bodies from stage 7,12, and 14 (mature oocyte) egg chambers expressing Me31B::GFP. Time constant (Tau), mobile, and immobile fractions of

Me31B estimated from recovery profiles of the whole FRAP (n = 9 for stage 7, n = 6 for stage 12, n = 16 for stage 14).

(B) Quantification of amplitude across P body recovery profiles from different stages of oogenesis reveals very little difference in the extent of recoveries FRAP (n = 9 for stage 7, n = 6 for stage 12, n = 16 for stage 14).

(C) Kymograph of *in vivo* P body from a mature oocyte expressing Me31B::GFP after whole FRAP shows no recovery, while kymograph of P bodies after internal FRAP displays recovery indicative of diffusion mediated recovery (whole FRAP n = 20, internal FRAP n = 24).

(D) Time constant (Tau), mobile, and immobile fractions of Me31B estimated from recovery profiles of whole FRAP or internal FRAP of P bodies from mature oocytes expressing Me31B::GFP (whole FRAP n = 20, internal FRAP n = 24).

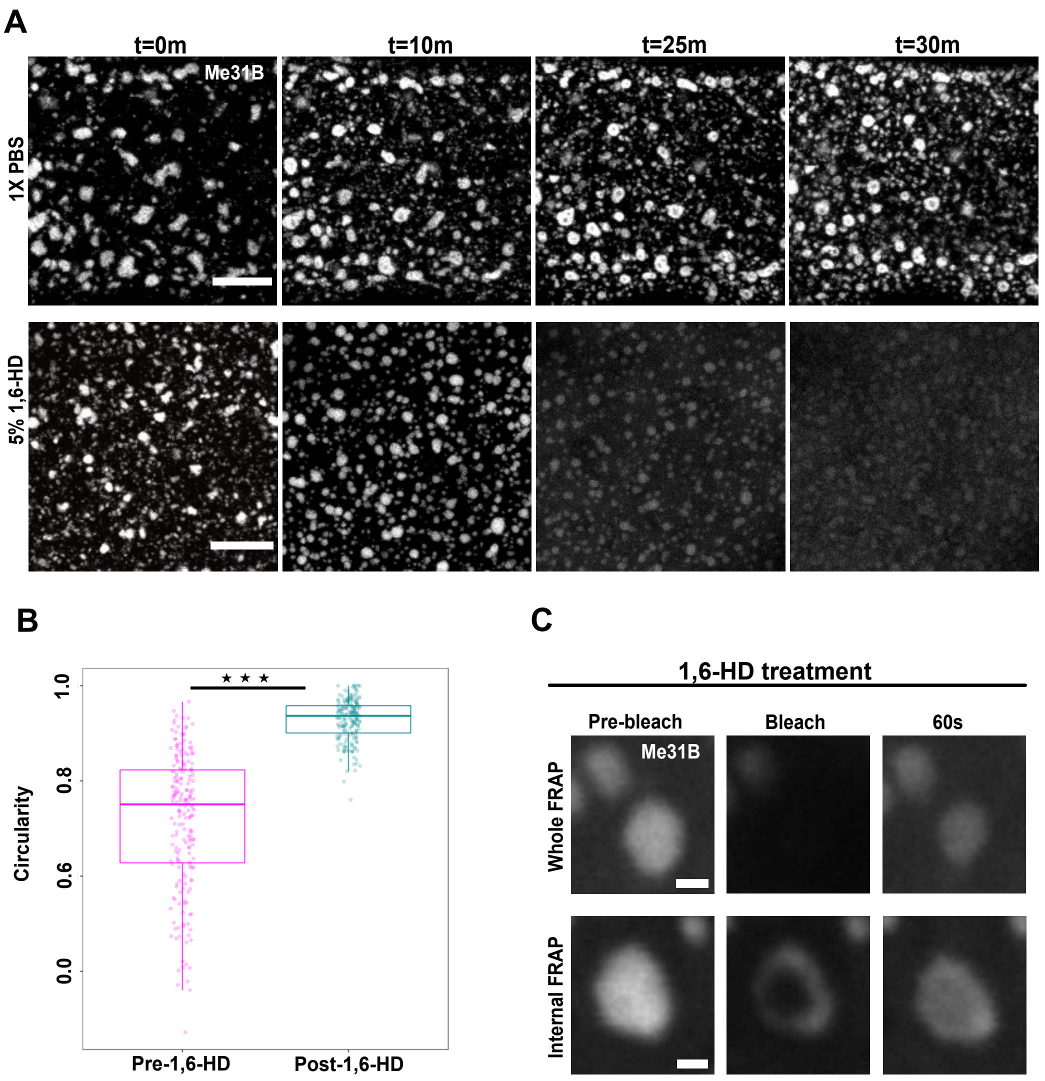

D

Experiment	Tau (seconds)	Mobile fraction	Immobile fraction
1,6-HD treated	10.16	0.641	0.358
Untreated	94.86	0.087	0.913

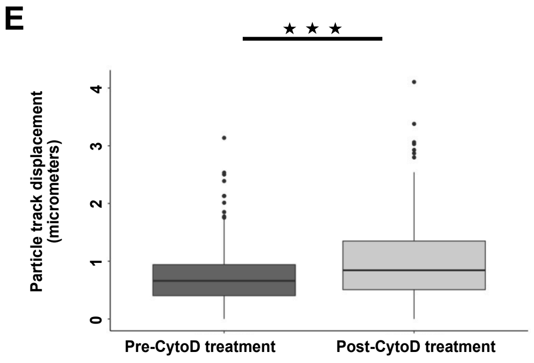

Figure S3. 1,6-HD and cytochalasin D treatments affect P body physical properties and dynamics. Related to Figure 3.

(A,C) Mature oocytes expressing Me31B::GFP.

(A) Time series of mature oocytes treated with 1X PBS (control) or 5% 1,6-HD shows that P bodies remain condensed in the control treatment while 1,6-HD leads to the dissolution of P bodies over time (n = 20 mature oocytes). Maximum projection 5 µm.
(B) Quantification of P body circularity prior to and after 1,6-HD treatment shows a significant increase in P bodies exhibiting spherical morphology post treatment (n = 200 P bodies, $p<0.0001$).
(C) Time series of P body condensates subjected to whole (n = 12) or internal (n = 3) FRAP after 1,6- HD treatment, both displaying rapid fluorescence recovery.
(D) Time constant (Tau), mobile, and immobile fractions of Me31B estimated from recovery profiles after being subjected to whole FRAP before or after treatment with 1,6-HD from mature oocytes expressing Me31B::GFP (n = 12).
(E) Quantification of particle displacement using TrackMate (Tinevez et al., 2017) software shows that P bodies in mature oocytes expressing Me31B::GFP after cytochalasin D treatment have increased spatial displacement compared to untreated P bodies (pre-treatment n = 674, post treatment n = 623 , $p<0.0001$).
Scale bar = 5 µm (A), 1 µm (C).

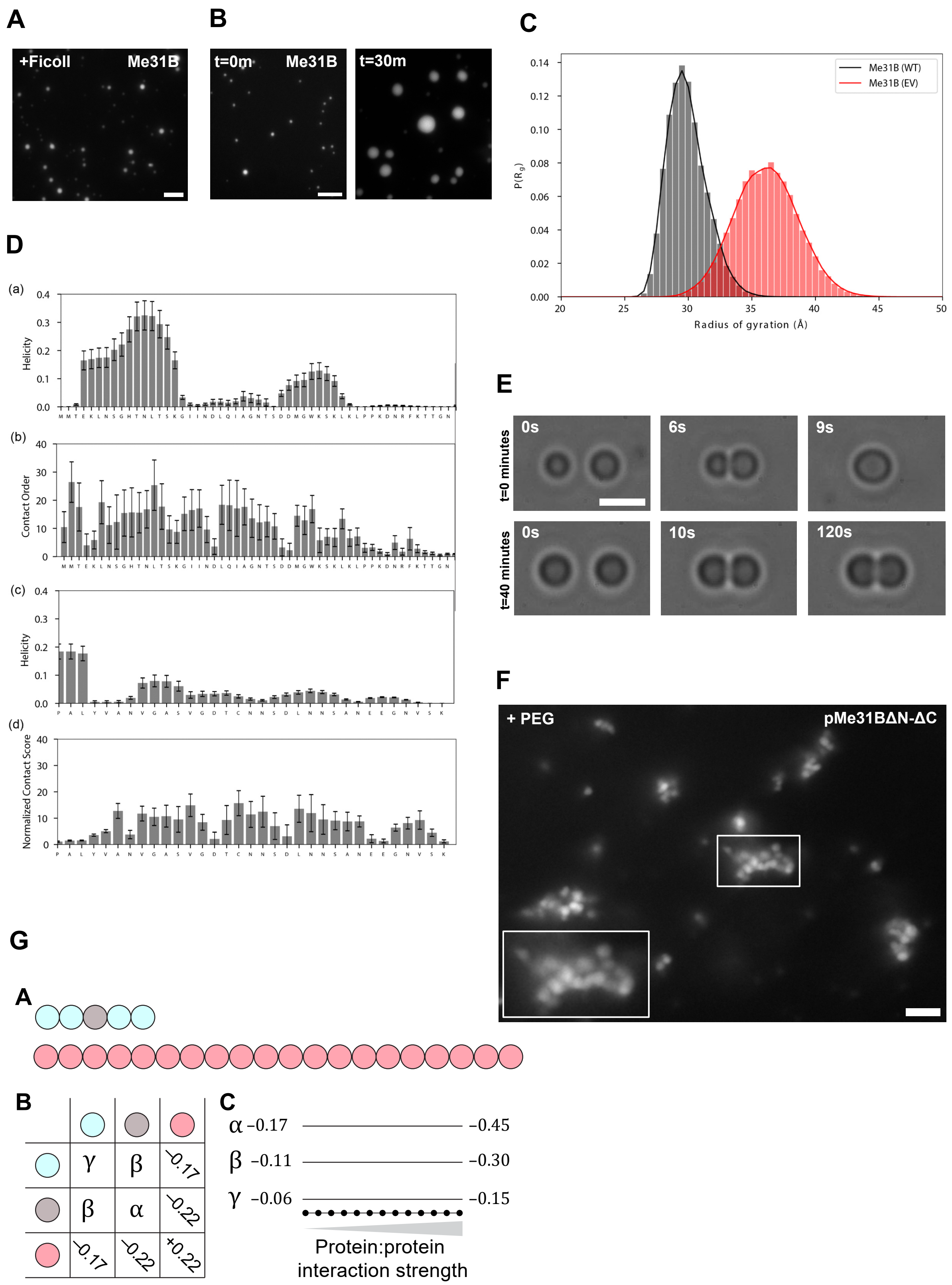

Figure S4. Interaction of IDRs with folded domains regulates Me31B physical state *in vitro*. Related to Figure 4 and Figure 6.

(A) 7.5 μm purified GFP-pMe31B in the presence of 1% Ficoll readily forms phase separated condensates (n = 10 independent replicates).

(B) Time series of purified GFP-pMe31B (7.5 μm) shows increase in size of condensates over 30 minutes (n = 10 independent replicates).

(C) Black bars show the Rg distribution for full-length Me31B under standard simulation conditions (full Hamiltonian (WT)), while red bars show the analogous distribution of the radius of gyration for simulations performed in which all attractive interactions are turned off (excluded volume (EV)). The full Hamiltonian simulations are substantially more compact, with an ensemble average radius of gyration of 30.1 Å, compared to 36.4 Å for the EV simulations. This compaction of the global dimensions originates from favorable interaction between the two IDRs and the folded domains.

(D) Local helicity (a,c) and intramolecular contacts (b,d) quantified on a per-residue basis for the NTD (a,b) and CTD (c,d). The NTD possess two short transient helices (4-17 and 31-37), while the CTD is entirely devoid of secondary structures. The NTD engages in more extensive intramolecular interactions than the CTD, as quantified by the normalized contact score (d) (see methods, larger values mean more contacts per residue). In both cases, the IDRs engage relatively uniformly, as opposed to via a specific motif. This implies broad and non-specific interactions between the IDRs and the folded domains.

(E) Time series of GFP-pMe31B condensate coalescence using optical traps. Rapid fusion of condensates is observed pre-gelation while condensates fail to fuse post-gelation (n = 25).

(F) pMe31B with both IDRs deleted (pMe31B ΔN-ΔC), in the presence of 1% PEG, fails to coalesce and rather forms amorphous aggregates (n = 10 independent replicates).

(G) Topology of Me31B (top) and RNA (bottom) molecules used in coarse-grained simulations (A)**.** Basic interaction table showing relative bead-bead interaction strengths with protein:protein bead interactions defined in terms of parameters (B). Protein:protein interaction parameters scale uniformly between max and min values (C).

Scale bar = 5 μm (A,B,F), 2 μm (E).

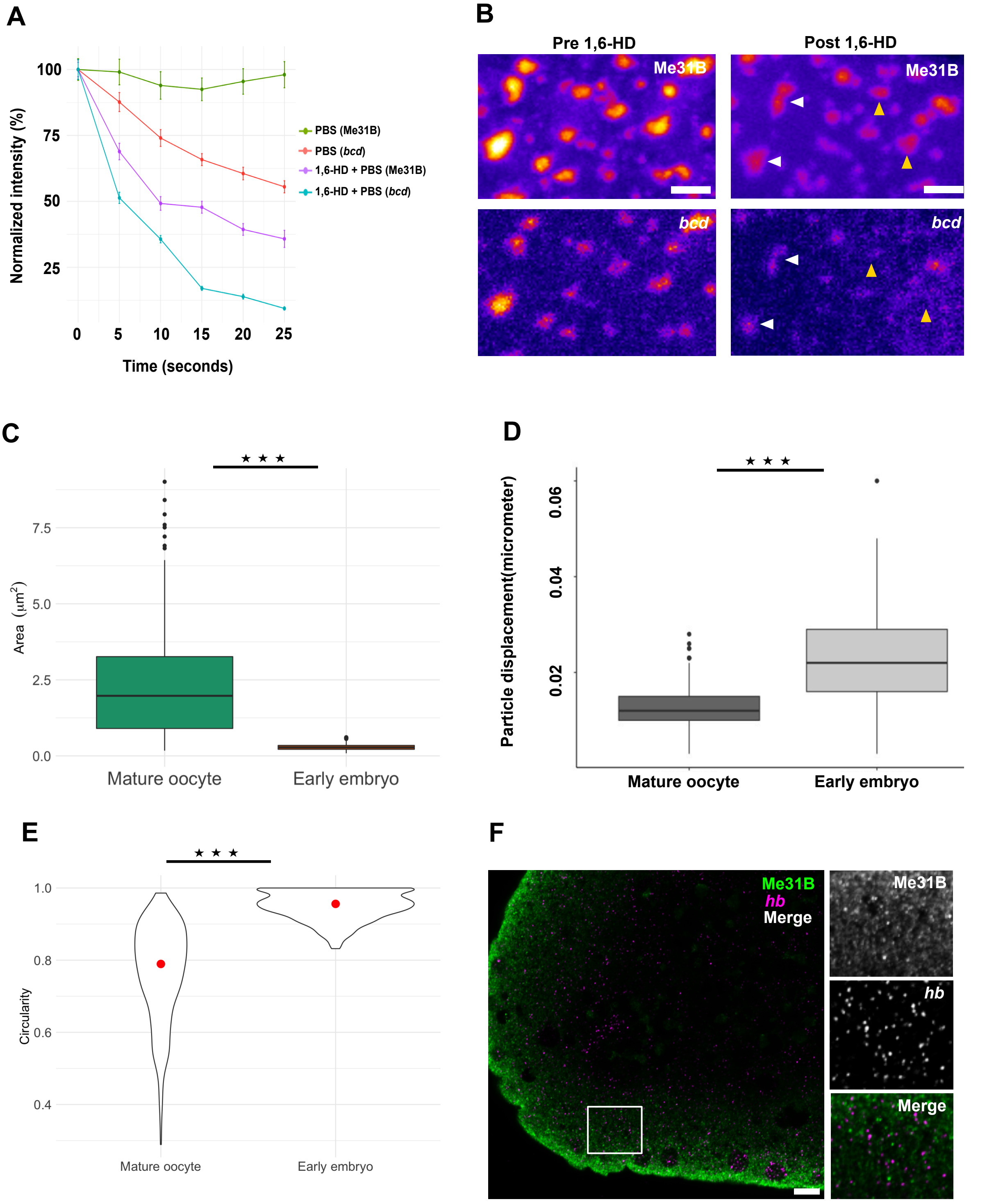

Figure S5. Egg activation modulates P body physical properties. Related to Figure 6.

(A) Quantification of images in Figure 6D and E. P body and *bcd* mRNA fluorescence in the presence of PBS (n = 35 Me31B and *bcd* particles) or 1,6-HD (n = 55 Me31B and *bcd* particles).

(B) Mature oocytes expressing Me31B::GFP, *hsp83-MCP-RFP,* and *bcd*-(*ms2*)$_6$ treated with 1,6-HD showing differential relationship of P bodies and *bcd* mRNA association (n = 55). In

some cases, *bcd mRNA* is retained in condensed P bodies post 1,6-HD addition (while arrowheads), while in others *bcd* is dispersed from condensed P bodies (yellow arrowhead).

(C) Quantification of P body sizes between mature oocytes and early embryos expressing Me31B reveals a significant decrease in embryonic P bodies (n = 400, p<0.0001).

(D) Quantification of particle displacement using TrackMate software shows that P bodies in the early embryo (n = 169) show increased track displacement compared to P bodies in the oocyte (n = 332, p<0.0001).

(E) Quantification of P body circularity from embryos expressing Me31B::GFP show that they are significantly more spherical compared to P bodies in the mature oocyte (n = 400, p<0.0001).

(F) Early embryo expressing Me31B::GFP labelled with GFP-Booster and FISH for *hb* mRNA shows no co-localization of P bodies and *hb*. Inset shows a zoomed in version of *hb* mRNA and P body distribution (n = 10 early embryos). Maximum projection 5 µm.

Scale bar = 2 µm (B), 5 µm (F).

**Symbol**	**Name**	**Fly Base ID**	**UniProt ID**
me31B	maternal expression at 31B	FBgn0004419	P23128
tral	trailer hitch	FBgn0041775	Q9VTZ0
bru1	bruno 1	FBgn0000114	O02374
cup	cup	FBgn0000392	Q9VMA3
DCP1	Decapping protein 1	FBgn0034921	Q9W1H5
DCP2	Decapping protein 2	FBgn0036534	Q5U127
edc3	Enhancer of decapping 3	FBgn0036735	Q9VVI2
eIF4E1	eukaryotic translation initiation factor 4E1	FBgn0015218	P48598
exu	exuperantia	FBgn0000615	P28750
lost	lost	FBgn0263594	Q9VN21
Patr-1	Protein associated with topo II related - 1	FBgn0266053	Q9VEN9
Hrb27C	Heterogeneous nuclear ribonucleoprotein at 27C	FBgn0004838	P48809
orb	oo18 RNA-binding protein	FBgn0004882	Q8IMZ2
pcm	pacman	FBgn0020261	Q9XZU2
stau	staufen	FBgn0003520	P25159
sqd	squid	FBgn0263396	Q08473
AGO3	Argonaute 3	FBgn0250816	Q7PLK0

Table S1. List of *Drosophila* P body proteins. Related to Figure 5A.

5'-GAAACTCTCTAACACGCCTC-3'
5'-ACAGTGGTTAACCTAAAGCT-3'
5'-TGGTATTTGTACAATCAGGA-3'
5'-CTTTCTACGCGTAGATATCT-3'
5'-ACGGATCTTAGGACTAGACC-3'
5'-AAACTTCCCTGGGAACCATT-3'
5'-CTGCTGACTAGGCTAGTACA-3'
5'-GATATGCACTGGAATCCGTG-3'
5'-GAGTTAACTGGAGTATCACT-3'
5'-AGCGTATTGCAGGGAAAGTA-3'
5'-CACCCAGATACATCTAAGGC-3'
5'-CATATTCCCGGGCTTTAGTG-3'
5'-TGGCCTCAAATGTAACTGGT-3'
5'-ACTTTCCATGGAATACGCTT-3'
5'-ATTTCCGAAATGTGGGACGA-3'
5'-AGAAGATTTTCTTGCTGGCT-3'
5'-GTACAGTTTTTAGCTATGTC-3'
5'-ATGAGATTACGCCCAAGAGA-3'
5'-ATGTTCGATCTTTAAGGGTA-3'
5'-ACACTTTGGCATAGCATAGA-3'
5'-GCGCAAATGTTTGATTATGT-3'
5'-TTGCTGACTATTCTTGGTCA-3'
5'-ACAAATGGTCTGCATTGATT-3'
5'-TGATAGTTATTCCGTTTGGC-3'
5'-ATGCTCTTCTTAGTGATGTA-3'
5'-ACTTGAGGCCTAACAGATTG-3'
5'-ACAACATCAAAGGTGCAGCA-3'
5'-ATTTACCCGAGTAGAGTAGT-3'

Table S2. Custom Stellaris® FISH Probes for the 3'UTR of *bcd* RNA. Related to Figure 6 and Figure 7 (adadpted from Trovisco et al., 2016).
