# Supplementary material for: Adaptable P body physical states differentially regulate bicoid mRNA storage during early Drosophila development
Source: Dev Cell. 2021 Oct 25;56(20):2886–2901.e6. doi: 10.1016/j.devcel.2021.09.021 (PMC8555633; doi:10.1016/j.devcel.2021.09.021)
Supplement: Document S2. Article plus supplemental information [file mmc2.pdf]

Developmental Cell

Article

Adaptable P body physical states differentially regulate *bicoid* mRNA storage during early *Drosophila* development

Graphical abstract

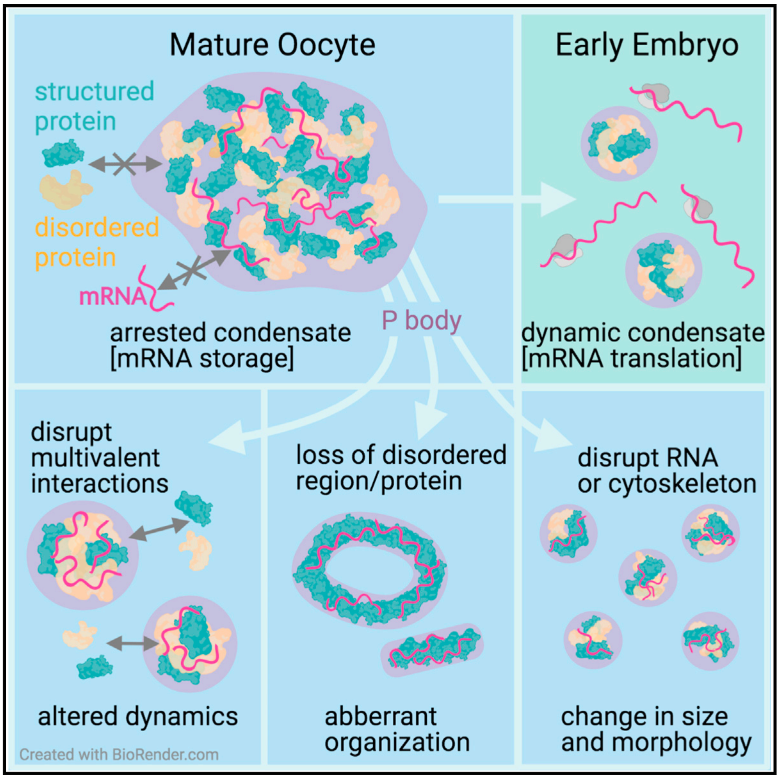

Authors

M. Sankaranarayanan, Ryan J. Emenecker, Elise L. Wilby, ..., Simon Alberti, Alex S. Holehouse, Timothy T. Weil

Correspondence

msnsankar18@gmail.com (M.S.), tw419@cam.ac.uk (T.T.W.)

In brief

Sankaranarayanan et al. show that P bodies, conserved ribonucleoprotein condensates found in *Drosophila* oocytes, are regulated by specific structural features and weak multivalent interactions. *In vivo*, P bodies adopt an arrested physical state that is critical for the storage of the *bicoid* patterning mRNA until egg activation.

Highlights

- P bodies adopt an arrested physical state in the mature oocyte
- Multivalent interactions and structurally distinct proteins regulate P body properties
- The arrested state and integrity of P bodies support the storage of *bicoid* mRNA
- Egg activation modulates P body properties and leads to release of *bicoid* mRNA

Sankaranarayanan et al., 2021, Developmental Cell *56*, 2886–2901
October 25, 2021 © 2021 The Authors. Published by Elsevier Inc.
https://doi.org/10.1016/j.devcel.2021.09.021

CellPress
OPEN ACCESS

Developmental Cell

Article

Adaptable P body physical states differentially regulate *bicoid* mRNA storage during early *Drosophila* development

M. Sankaranarayanan,[1,*] Ryan J. Emenecker,[2,3] Elise L. Wilby,[1] Marcus Jahnel,[4] Irmela R.E.A. Trussina,[4] Matt Wayland,[1] Simon Alberti,[4] Alex S. Holehouse,[2,3] and Timothy T. Weil[1,5,*]
[1]Department of Zoology, University of Cambridge, Downing Street, Cambridge CB2 3EJ, UK
[2]Department of Biochemistry and Molecular Biophysics, Washington University School of Medicine, 660 S. Euclid Ave., St. Louis, MO 63110, USA
[3]Center for Science and Engineering of Living Systems, Washington University in St. Louis, 1 Brookings Drive, St. Louis, MO 63130, USA
[4]Center for Molecular and Cellular Bioengineering, Biotechnology Center, Technische Universität Dresden, Tatzberg 47/49, 01307 Dresden, Germany
[5]Lead contact
*Correspondence: msnsankar18@gmail.com (M.S.), tw419@cam.ac.uk (T.T.W.)
https://doi.org/10.1016/j.devcel.2021.09.021

SUMMARY

Ribonucleoprotein condensates can exhibit diverse physical states *in vitro* and *in vivo*. Despite considerable progress, the relevance of condensate physical states for *in vivo* biological function remains limited. Here, we investigated the physical properties of processing bodies (P bodies) and their impact on mRNA storage in mature *Drosophila* oocytes. We show that the conserved DEAD-box RNA helicase Me31B forms viscous P body condensates, which adopt an arrested physical state. We demonstrate that structurally distinct proteins and protein-protein interactions, together with RNA, regulate the physical properties of P bodies. Using live imaging and *in situ* hybridization, we show that the arrested state and integrity of P bodies support the storage of *bicoid (bcd)* mRNA and that egg activation modulates P body properties, leading to the release of *bcd* for translation in the early embryo. Together, this work provides an example of how physical states of condensates regulate cellular function in development.

INTRODUCTION

Many biochemical reactions in the cytoplasm of eukaryotic cells require regulation in space and time. The organization of specific reactions in the dense cytoplasmic environment is achieved through membrane-bound and membrane-less organelles. Classic membrane-bound organelles, such as the nucleus and endoplasmic reticulum, are stable micro-environments that are enclosed by membranes. However, membrane-less organelles, such as stress granules, P bodies, and nuclear bodies, which are typically composed of nucleic acids and proteins, have been shown to provide an additional level of cellular organization (Buchan and Parker, 2009; Banani et al., 2017; Shin and Brangwynne, 2017; Boeynaems et al., 2018). More generally, the designation *biomolecular condensates* is used to describe cellular assemblies characterized by the non-stoichiometric concentration of biomacromolecules, of which membrane-less organelles are one such example (Brangwynne et al., 2009; Li et al., 2012; Hubstenberger et al., 2013; Wang et al., 2014; Nott et al., 2015; Zhang et al., 2015; Feric et al., 2016; Hyman et al., 2014; Brangwynne et al., 2015; Banani et al., 2017; Lyon et al., 2021).

Ribonucleoprotein (RNP) complexes are an abundant and conserved class of biomolecular condensates. Found both in the cytoplasm and the nucleus, RNP condensates exhibit a wide range of physical states, ranging from dynamic liquids to stable solids (Kroschwald et al., 2015, 2018; Weber, 2017; Weber and Brangwynne, 2012; Woodruff et al., 2017). Some examples of diverse physical states observed *in vivo* include liquid-like P granules in *Caenorhabditis elegans (C. elegans)* embryos (Brangwynne et al., 2009; Wang et al., 2014), viscous nucleoli in *Xenopus laevis* oocytes (Brangwynne et al., 2011; Feric et al., 2016; Mitrea et al., 2016), and solid-like Balbiani bodies vertebrate oocytes (Boke et al., 2016). Despite considerable progress, the relationship between the physical states of biomolecular condensates and their *in vivo* function remains poorly understood.

RNP condensates are often linked with localized RNA translational regulation, which enables cells to spatiotemporally regulate protein synthesis (Medioni et al., 2012). Specialized cells, including neurons and oocytes, frequently depend on this mode of post-transcriptional regulation to control gene expression (Jung et al., 2014; Kloc and Etkin, 2005). More specifically, transcriptionally inactive oocytes, such as those in *Drosophila melanogaster*, rely on prolonged storage and translational control of maternally deposited transcripts for body axes patterning during development (Lasko, 2012; Tadros and Lipshitz, 2009).

This is an open access article under the CC BY license (http://creativecommons.org/licenses/by/4.0/).

One mechanism for regulating RNA metabolism involves P bodies, an evolutionarily conserved class of cytoplasmic biomolecular condensates (Sheth and Parker, 2003; Andrei et al., 2005; Kedersha et al., 2005; Eulalio et al., 2007; Parker and Sheth, 2007; Buchan, 2014; Hubstenberger et al., 2017; Luo et al., 2018). Previous studies on P bodies have highlighted their roles in RNA storage and translational repression, since P bodies are devoid of ribosomes (Hubstenberger et al., 2017; Weil et al., 2012). A conserved component of P bodies is the ATP-dependent DEAD-box RNA helicase: DDX6 in humans, Dhh1 in yeast, CGH-1 in *C. elegans,* and Maternal expression at 31B (Me31B) in *Drosophila*. Me31B is required for early *Drosophila* development and is estimated to be present at concentrations of ~7.5 μM in the egg (Götze et al., 2017). During oogenesis, Me31B is known to associate with and differentially regulate several axis-patterning maternal mRNAs (Nakamura et al., 2001; Weil et al., 2012; Kaneuchi et al., 2015; Tadros and Lipshitz, 2009; York-Andersen et al., 2015). Failure to regulate these and many other mRNAs can lead to severe developmental defects (Lasko, 2012). However, the mechanisms that underlie how transcripts are maintained and translationally controlled by P bodies are not well understood.

To examine the *in vivo* basis of mRNA regulation, we employ a multidisciplinary approach to investigate the physical properties and *in vivo* functions of P bodies in mature *Drosophila* oocytes. Real-time live imaging reveals that P body condensates adopt a highly viscous and arrested physical state in mature oocytes, and their integrity depends on electrostatic and hydrophobic interactions, along with RNA and the actin cytoskeleton. Using *in silico, in vitro*, and *in vivo* assays, we demonstrate that intrinsically disordered regions (IDRs) in Me31B and the disordered P body protein Trailer hitch (Tral) independently regulate the assembly and physical properties of Me31B condensates. Using live imaging and single-molecule fluorescent *in situ* hybridization (smFISH), we demonstrate that the arrested state and integrity of P bodies is critical for the storage of *bcd* mRNA, which is later released for translation at egg activation. Finally, we show that P bodies in the early embryo are smaller and highly dynamic than in the oocyte, and do not co-localize with translationally active *bcd* mRNAs. Together, our results highlight an *in vivo* role for adaptable P body physical states in mRNA regulation during development.

RESULTS

Me31B forms viscous P body condensates, which adopt an arrested physical state in mature oocytes

Several maternal mRNAs are thought to be stored and regulated by P bodies throughout *Drosophila* oogenesis (Lin et al., 2008; Nakamura et al., 2001; Weil et al., 2012). However, a mechanistic understanding of how P bodies accomplish this function remains unclear. To examine the physical state of P bodies, we isolated living-stage 14-egg chambers (hereafter referred to as mature oocytes) from female *Drosophila* (Figure 1A). Live imaging of Me31B::GFP revealed that P bodies are typically micron-sized condensates with varying morphologies (Figures 1B and 1C). We also found that most P bodies have internal subdomains suggestive of a heterogeneous organization (Figure 1D). Quantification of P body aspect ratios over time showed that P bodies have predominantly irregular morphologies (Figure 1E) compared with liquid-like condensates, such as P granules and stress granules (Brangwynne et al., 2009; Patel et al., 2015). Time-lapse imaging also demonstrated that P bodies undergo continuous rearrangements, mostly progressing from amorphous to spherical morphologies, as exemplified by their aspect ratio analysis over the observed timescales (Figure 2A). Additionally, P bodies undergo fusion and fission events, which are hallmarks of a dynamic state (Figures 2B, 2B′, and S1). However, the longer timescale of these events suggests that P bodies in mature *Drosophila* oocytes are less dynamic compared with liquid-like condensates.

The cytoplasm of mature oocytes is packed with yolk granules and complex cytoskeletal structures. To determine if the slow P body dynamics are an intrinsic property or dependent on the oocyte cytoplasmic environment, we developed an *ex vivo* assay, whereby we extruded the cytoplasm into halocarbon oil (Figure 2C). Importantly, this approach does not promote P body dissolution, rather, extruded P bodies initially exhibit irregular morphologies but become spherical over the observed timescale (Figure 2C′). We also show that the extruded P bodies undergo fusion and fission events (Figures 2D and 2D′), but these are faster than *in vivo*, likely due to the absence of cytoplasmic crowding and cytoskeletal structures. Taken together, our data suggest that P bodies are slowly rearranging condensates, and that this physical property is inherent to P bodies in mature *Drosophila* oocytes.

Next, we performed fluorescence recovery after photobleaching (FRAP) on whole P bodies (whole FRAP) to examine the mobility of Me31B between the cytoplasm and the P body. This analysis revealed that Me31B localized to P bodies exhibited limited or no recovery (Figures 2E and 2F). To assess if this is a general property of P bodies, we performed whole FRAP of P bodies in earlier stages of oogenesis. The recovery patterns and the proportion of immobile Me31B were similar to those observed in mature oocytes (Figures S2A and S2B). Due to the limited exchange of Me31B between P bodies and the cytoplasm, we refer to this as the arrested state of P bodies.

To further explore Me31B dynamics, we tested if Me31B can rearrange within P bodies by assessing the mobility of Me31B after photobleaching within a region inside the P body (internal FRAP) (Figures 2E and 2F). Measurements revealed considerable recovery of fluorescence compared with whole FRAP (Figure S2C). Despite a high mobile fraction, the rate of recovery indicates that the dynamics of Me31B within P bodies is slow (Figure S2D). We further derived an apparent viscosity in the range of ~700 Pa.s for P bodies from their internal recovery kinetics. Although this estimate should be treated qualitatively, the value is at least two orders of magnitude larger than those reported for liquid-like condensates (Alshareedah et al., 2021; Brangwynne et al., 2009). Overall, these data show that P bodies in mature oocytes adopt a viscous and arrested physical state.

Multivalent interactions, RNA, and the actin cytoskeleton regulate P body physical properties

Previous work has shown that activation of the mature oocyte results in an influx of monovalent and divalent ions, release of stored mRNAs, and reorganization of the actin cytoskeleton (York-Andersen et al., 2015, 2020; Kaneuchi et al., 2015).

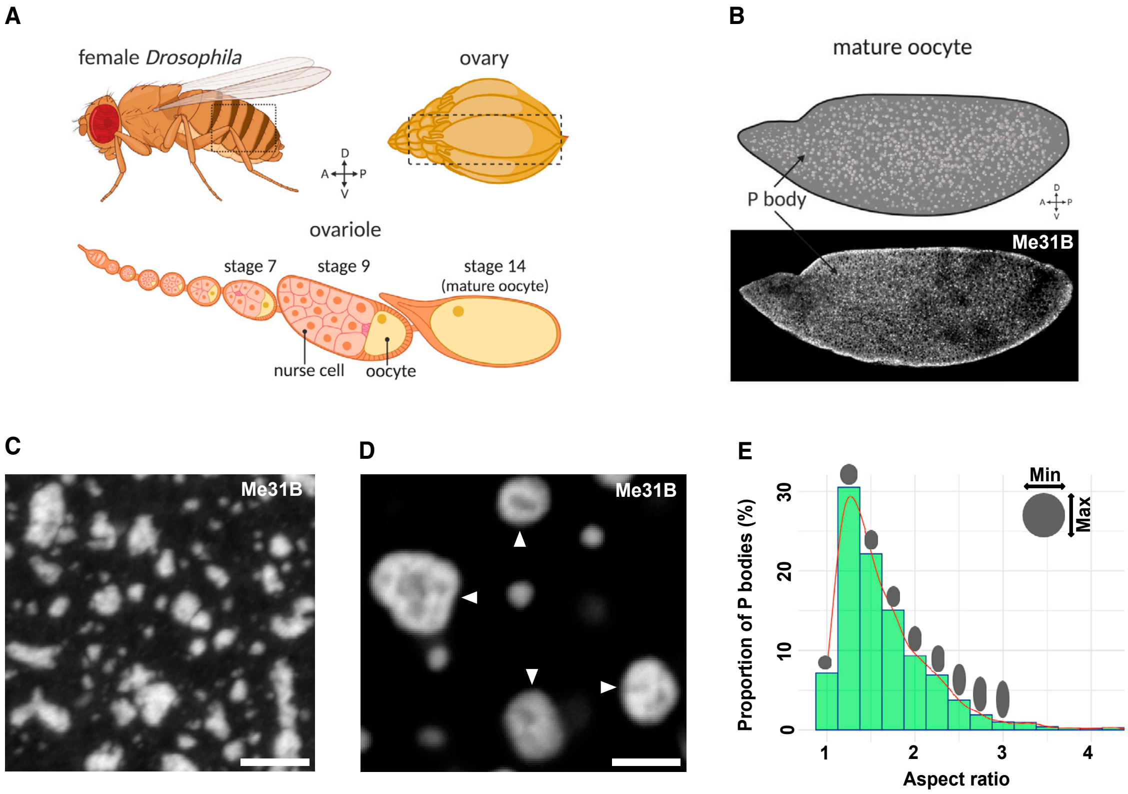

Figure 1. Me31B forms heterogeneous P body condensates in the mature oocyte

(A) Schematic of a *Drosophila* female ovary and ovariole. Each female contains two ovaries, each comprising 16–18 ovarioles. Each ovariole can be thought of as an assembly line for the production of mature oocytes. The oocyte is supported by a collection of nurse cells until the late stages of oogenesis. Created with BioRender.com.

(B–E) Mature oocyte (~ 0.5 mm in length) expressing Me31B::GFP.

(B) Cartoon depicting P body distribution in the mature oocyte and confocal image of a whole mature oocyte showing P bodies throughout the cytoplasm. The concentration of P bodies at the cortex is, in part, due to this being a cross section image.

(C) Increased magnification of P bodies in the mid-lateral area of the oocyte reveals they exhibit diverse morphologies and sizes. Maximum projection 10 μm.

(D) Representative image of P bodies exhibiting multiple subdomains (white arrowheads) indicative of heterogeneous internal organization.

(E) Aspect ratio analysis of individual P bodies (>1 μm) showing an uneven range of P body morphology (n = 20). Scale bar, 5 μm (C), 2 μm (D).

Therefore, we wondered if these factors could regulate the physical properties of P bodies in the mature oocyte prior to egg activation. Various interactions have been shown to contribute to RNP condensation, including hydrophobic and electrostatic interactions (Brangwynne et al., 2015; Dzuricky et al., 2020; Kato et al., 2012; Murthy et al., 2019; Nott et al., 2015; Pak et al., 2016; Riback et al., 2017). The interactions that are thought to drive P body assembly can be interpreted through the lens of a pseudo two-component phase diagram (Figure 3A). In particular, by changing the solution conditions to weaken the interactions that contribute to P body assembly, theory and simulations predict an increase in internal mobility and more spherical shaped condensates, as shown previously for protein-RNA condensates (Boeynaems et al., 2019).

To test if hydrophobic interactions are required for *in vivo* P body integrity, we treated mature oocytes with the aliphatic alcohol 1,6-hexanediol (1,6-HD), a compound identified originally in the context of attenuating hydrophobic interactions (Ribbeck and Görlich, 2002; Patel et al., 2007). The addition of 1,6-HD resulted in the transformation of P bodies toward a more spherical shape, an increase in fusion events, and, ultimately, the dissolution of condensates over time (Figures 3B, 3C, S3A, and S3B). These results support a model in which 1,6-HD weakens the multivalent interactions that contribute to P body physical state and integrity. To further test if 1,6-HD leads to a transition from an arrested to a more dynamic state, we performed whole FRAP on 1,6-HD-treated P bodies. Consistent with our model, P bodies exhibited rapid and sustained recovery (Figures 3D, S3C, and S3D). Although 1,6-HD-treated P bodies only exhibited appreciable recovery up to ~60%, this is likely due to their dissolution occurring simultaneously. Although 1,6-HD has been shown to affect mechanical properties in cultured cells, we did not observe any noticeable phenotypes in the mature oocytes over the observed timescales (Wheeler et al., 2016). Taken together, our results suggest that hydrophobic interactions contribute to regulating the arrested state of P bodies, which, in turn, maintains their integrity.

Next, we examined if electrostatic interactions contribute to P body physical properties by testing the impact of monovalent (NaCl) or divalent salts ($MgCl_2$). At low concentrations of

CellPress
OPEN ACCESS

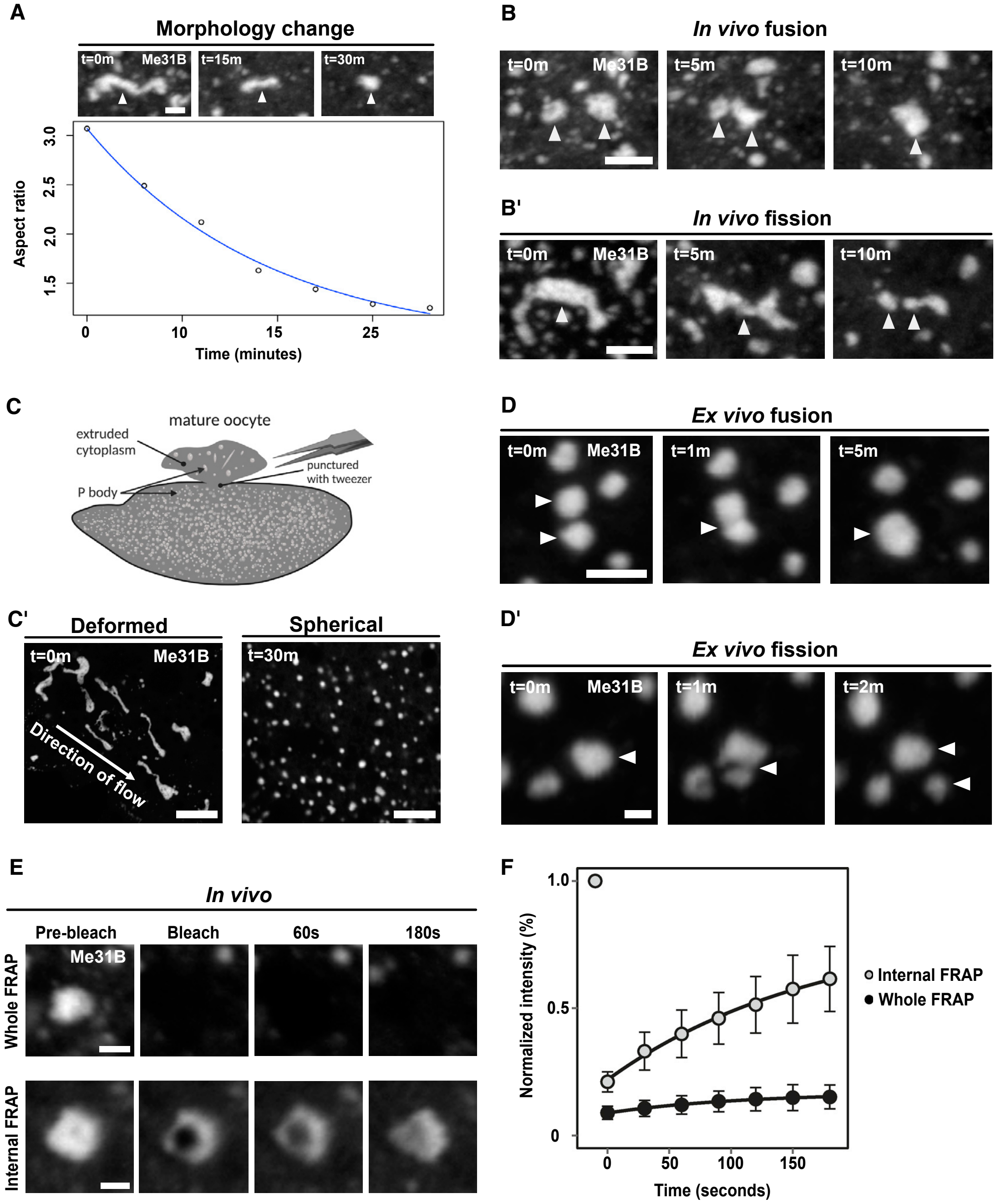

Figure 2. P bodies adopt a less dynamic and arrested physical state

(A–E) Mature oocyte expressing Me31B::GFP.

(A) Time series of a P body displaying elastic behavior, starting in an extended state (t = 0 min) and subsequently relaxing toward a spherical morphology (t = 30 min). Plot of individual P body (n = 10) A.R over time showing relaxation from extended (A.R~3) to spherical morphology (A.R~1).

(B) Time series of two *in vivo* P bodies undergoing coalescence (white arrowheads) (n = 20).

(B′) Time series of a single *in vivo* P body undergoing fission to form two distinct condensates (white arrowheads) (n = 20).

(C) Cartoon depicting cytoplasmic extrusion of P bodies into halocarbon oil (*ex vivo*) induced by puncturing the outer membrane of the mature oocyte. Created with BioRender.com.

(C′) *Ex vivo* P bodies displaying stretched elastic morphologies shortly after extrusion (t = 0 min). Over time, extruded P bodies relax into homogeneous spherical condensates (t = 30 min, n = 25).

(D) Time series of *ex vivo* P bodies undergoing coalescence (white arrowheads) (n = 20).

(D′) Time series of *ex vivo* extruded P bodies undergoing fission (white arrowheads) (n = 5).

(E) Time series of whole FRAP of P body shows minimal recovery, whereas internal FRAP of P body shows increased recovery of Me31B fluorescence.

(F) P body recovery profiles after whole FRAP (n = 20) and internal FRAP (n = 24) (mean, standard deviation).

Scale bar, 2.5 μm (A–D′), 10 μm (C′), 1.5 μm (E).

See also Figures S1 and S2.

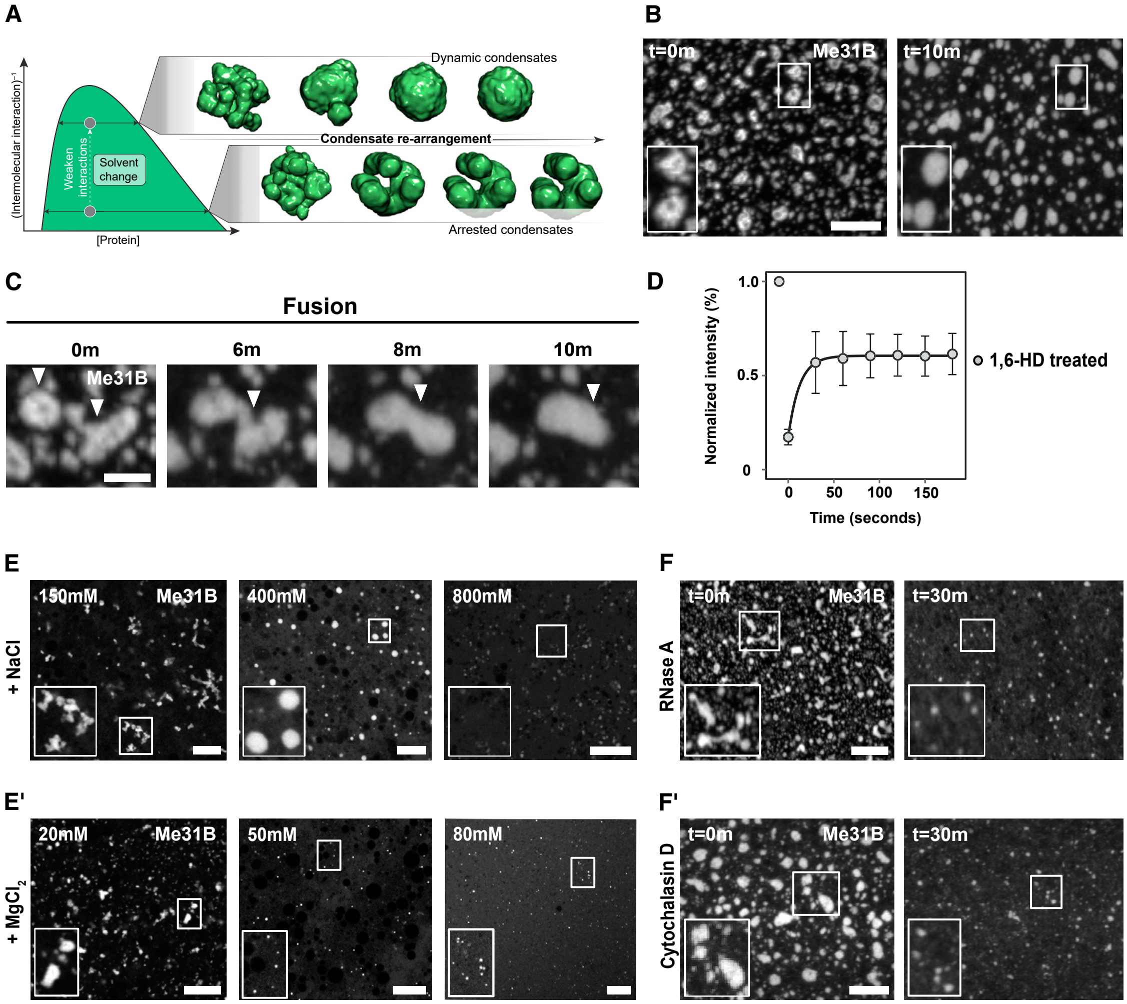

Figure 3. P body physical properties are regulated by hydrophobic and electrostatic interactions along with RNA and actin

(A) Schematized phase diagram in which protein concentration extends along the x axis, whereas molecular interaction strength extends across the y axis. Inset shows snapshots from coarse-grained simulations performed at distinct positions along the y axis.

(B, C, and E–F′) Mature oocyte expressing Me31B::GFP.

(B) The addition of 5% 1,6-HD causes P bodies to transform from amorphous to spherical morphology within 10 min and results in the loss of internal heterogeneity. Maximum projection 5 μm.

(C) Time series shows two P bodies undergoing coalescence following the addition of 1,6-HD (n = 30). Maximum projection 5 μm.

(D) Whole FRAP recovery profile of 1,6-HD-treated P bodies showing rapid fluorescence recovery (n = 12) (mean, standard deviation).

(E) The addition of varying concentrations of NaCl results in diverse physical states of extruded P bodies ranging from sticky (150 mM) to liquid-like (400 mM) and diffuse state (800 mM). Single plane image.

(E′) Treatment with $MgCl_2$ results in the dissociation of extruded P bodies at concentrations significantly lower than NaCl. Single plane image.

(F) Treatment with 500 ng/μl RNase A) or (F′) 10 μg/μl cytochalasin- D (depolymerizes actin) causes P body dissociation, resulting in smaller condensates. Maximum projection 10 μm.

n = 5 experimental repeats for (E–F').

Scale bar, 5 μm (B, C, and E–F′).

See also Figure S3.

NaCl, P bodies assemble into clusters, whereas at high concentrations they dissociate (Figure 3E). However, at intermediate concentration ranges (300–600 mM), P bodies adopt spherical morphologies, consistent with a more dynamic state. These results are supportive of a model in which electrostatic interactions, like hydrophobic interactions, play a role in dictating physical properties and can be tuned up or down by decreasing or increasing the monovalent salt concentrations, respectively.

Interestingly, the addition of 20 mM $MgCl_2$ had no apparent effect on P body integrity; yet, a small increase in concentrations as low as 50 mM $MgCl_2$ resulted in their complete dissociation (Figure 3E′). This relative sensitivity to divalent cations implies an effect beyond simply ionic strength. Collectively, these data suggest that changes in salt concentration can alter P body integrity, consistent with the morphology and state of P bodies that we observe following

CellPress
OPEN ACCESS

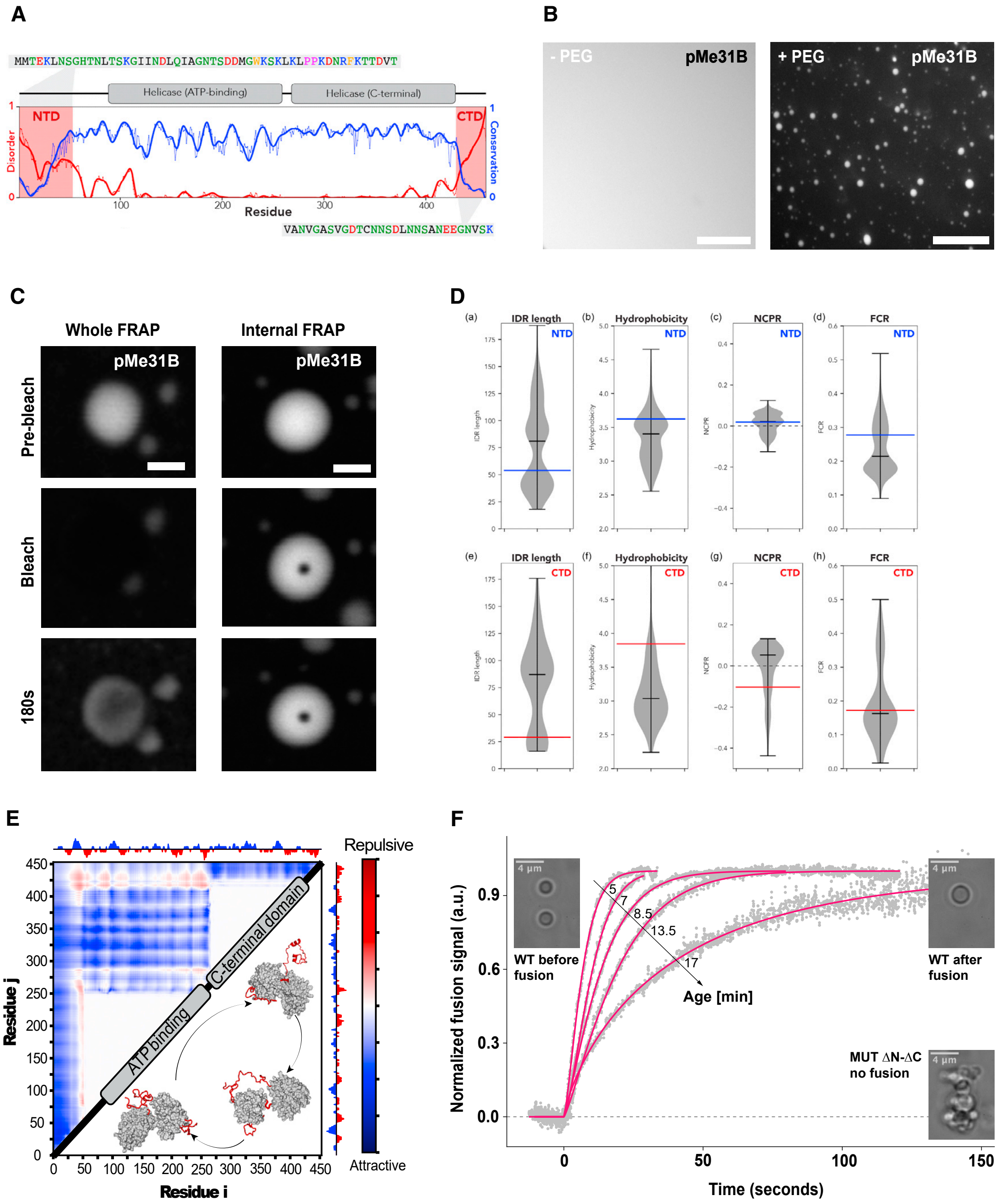

Figure 4. Deletion of IDRs results in aggregate-like Me31B condensates *in vitro*

(A) Overview of disordered, conservation, and domain architecture for Me31B. Conservation calculated across 566 orthologous sequences. N-terminal domain (NTD) and C-terminal domain (CTD), sequences are highlighted, with an atomistic model of the full-length protein shown in panel E.

(B) Purified GFP-Me31B (pMe31B) at 7.5 μM is diffuse on its own but forms phase separated spherical condensates in the presence of 1% PEG (n = 10). Maximum projection 5 μm.

(C) Time series of pMe31B condensates subjected to FRAP experiments. Whole P body photobleaching shows moderate fluorescence recovery, whereas internal FRAP shows no recovery (n = 10).

(D) Violin plots quantify density of IDR length (A/E), hydrophobicity (B/F), net charge per residue, (C/G) and fraction of charged residues (D/H) for the N-terminal IDRs (A–D) or C-terminal IDRs (E-H). Blue or red bars define the associated value for the Me31B IDR in the N- or C-terminal IDR, respectively.

(E) Summary of all-atom simulations. Normalized inter-residue distance is shown with cooler colors reflecting attractive interactions and warmer colors reflecting repulsive interactions.

(legend continued on next page)

ex vivo egg activation or in the early embryo (York-Andersen et al., 2015).

Given the importance of electrostatic interactions, we asked if P body integrity was regulated exclusively by protein-protein interactions, or if protein-RNA interactions also contributed. Previous biochemical studies have shown that P body proteins differentially interact with Me31B in an RNA-dependent or -independent manner (Nakamura et al., 2004). To test the effect of RNA on P body integrity, we treated mature oocytes with RNase A, which leads to P body dissociation into smaller-sized condensates (Figure 3F). The incomplete dissolution implies that *in vivo* P body integrity is largely dependent on protein-protein interactions, but this does not exclude a contribution from protein-RNA interactions.

Finally, to examine the role of actin in regulating P body integrity, mature oocytes were treated with cytochalasin D, a commonly used actin depolymerizing agent. This treatment resulted in the dissociation of P bodies in 30 min, consistent with our data from *ex vivo* egg activation (Figure 3F′). Since the actin cytoskeleton is commonly involved in RNP anchoring, we tested if the dissociated P body particles exhibited altered spatial dynamics (Medioni et al., 2012; Weil et al., 2008). Particle displacement analysis showed that cytochalasin-D-treated P bodies were significantly more mobile than untreated ones (Figure S3E). Taken together, these results indicate that multiple factors regulate P body integrity, properties, and dynamics in the mature oocyte.

IDRs regulate the physical state of Me31B condensates *in vitro*

Having identified multiple external factors in the regulation of P body integrity, we next asked if sequence features within the Me31B protein may be regulating P body physical state. Me31B contains an ATP-binding and folded helicase domain, flanked by short N- and C-terminal IDRs (Figure 4A). Although the function of the helicase domain is well studied, much less is known about the function of the disordered regions. Since Me31B is an essential *in vivo* protein, we adopted an *in vitro* approach to examine the role of these disordered regions.

We first tested if the purified recombinant Me31B (GFP-Me31B) can undergo condensation *in vitro*. Although Me31B is diffuse at physiological protein concentrations (7.5 μM), upon addition of a crowding agent, which mimics the oocyte cytoplasmic environment (1% PEG), Me31B formed spherical condensates (Figure 4B). We repeated this experiment using an alternative crowder (1% Ficoll) and confirmed that Me31B condensation does not depend on the specific chemical properties of the crowding agent (Figure S4A). Time-lapse imaging revealed that Me31B forms micron-sized spherical condensates, suggestive of a liquid-like state (Figure S4B). To examine Me31B mobility, we performed both whole FRAP and internal FRAP on freshly formed condensates. To our surprise, these condensates showed little or no recovery after photobleaching, indicating that Me31B condensates are present in an arrested physical state similar to *in vivo* P bodies (Figure 4C).

Next, we wondered what role IDRs might have in Me31B condensation. Previous work has shown that IDRs in DEAD-box helicases can contribute to RNP condensate formation and physical state, a property determined by sequence composition and length (Elbaum-Garfinkle et al., 2015; Hondele et al., 2019; Nott et al., 2015). We assessed conservation across a set of DDX6 orthologs (including Me31B), revealing that folded domains are highly conserved, whereas IDR length and sequence varied substantially (Figure 4D). Taken broadly, our results imply that Me31B and its orthologs may show differences in condensate formation tuned by their IDRs.

To better understand how the IDRs might contribute to function, we performed all-atom simulations of full-length Me31B, which revealed that both IDRs adopt a heterogeneous ensemble of states (Figure 4E). Interestingly, both N- and C-terminal IDRs interacted transiently and relatively non-specifically with the surface of the folded domains. These contacts were mediated through electrostatic and hydrophobic interactions (Figures S4C and S4D). Rather than acting as drivers of self-assembly, our simulations suggest the possibility that IDRs play a modulatory role.

To test for the modulatory influence of IDRs, we purified recombinant Me31B with the complete N- and C-terminal IDRs deleted (Me31BΔN-ΔC). We then used dual-trap optical tweezers to quantitatively measure the rate of condensate fusion events, thus providing a readout of their physical properties (Jahnel et al., 2011). We show that full-length Me31B condensates initially exhibit rapid fusion events; however, these decrease over time (Figure S4E). In contrast, Me31BΔN-ΔC condensates rapidly self-assembled into aggregate-like structures (Figures 4F and S4F). These results demonstrate that the IDRs tune the physical properties of Me31B condensates by attenuating the strong interactions established among the interacting folded domains.

Tral is key to regulating organization of P bodies in the mature oocyte

In addition to Me31B, several other proteins localize to or are found to be enriched within P bodies (Lin et al., 2008). Given the importance of disordered regions within Me31B, we hypothesized that the many IDRs found in P body proteins could potentially act as lubricants to regulate P body assembly and organization through interactions with structured proteins. To test this, we first performed disorder prediction across the set of known P body proteins to estimate the proportion of structured versus disordered regions (Figure 5A). Approximately 50% of all residues found within P body proteins are predicted to be disordered, highlighting the structural heterogeneity of components within P bodies. Among the proteins enriched with intrinsic disorder is Tral, a member of the LSM protein family (RAP55 in vertebrates, CAR-1 in *C. elegans*), which is known to interact directly with Me31B, function in *Drosophila* axis patterning, and is predicted to be largely disordered with the exception of an N-terminal LSM domain (Figure 5B) (Bouveret et al., 2000;

(F) Fusion of pMe31B condensates (magenta) at different time points post condensation, quantified by dual-TRAP optical tweezers. pMe31B ΔN-ΔC condensates (dashed line) do not fuse and rapidly aggregate with each other (n = 20).
Scale bar, 5 μm (B), 1 μm (C).
See also Figure S4.

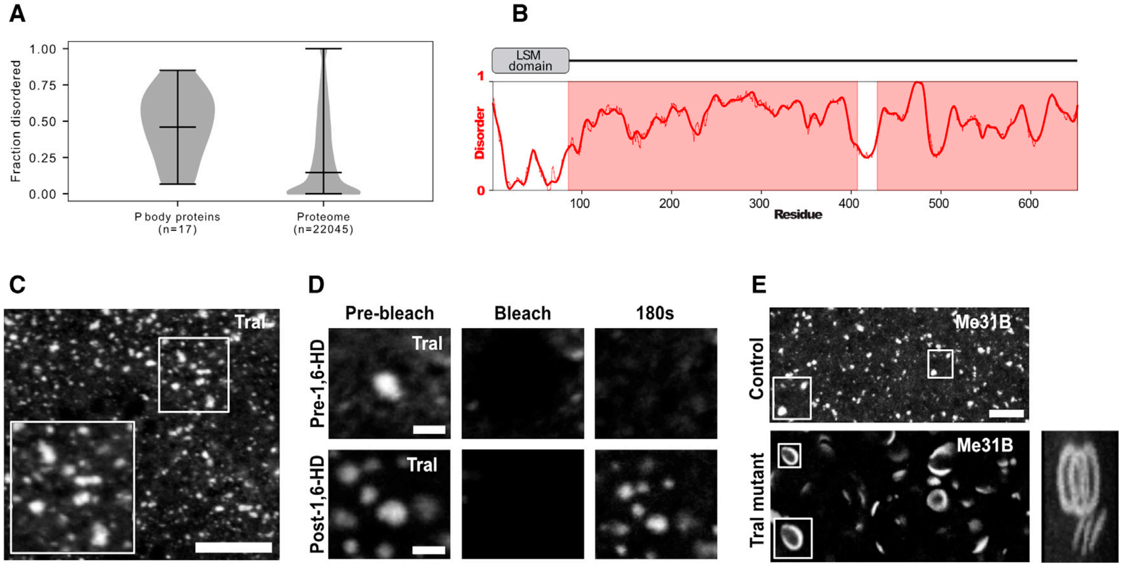

Figure 5. Absence of Tral alters P body morphology in the mature oocyte

(A) Comparison of fraction disorder in known *Drosophila* P body proteins (left) compared with whole *Drosophila* proteome (right). The average fraction disorder of the 17 proteins (Table S1) associated with the P body is over 99.9% more disordered than any possible random sized-matched set of proteins taken from the *D. melanogaster* proteome.

(B) Schematic of Tral domain architecture containing a structured LSM domain followed by a long stretches of highly disordered regions.

(C and D) Mature oocyte expressing GFP::Tral.

(C) Tral localizes to P body condensates with diverse morphologies and sizes, distributed throughout the oocyte cytoplasm (n = 20). Maximum projection 7 μm.

(D) Time series of FRAP experiments on GFP::Tral condensates before and after treatment with 1,6-HD (n = 13).

(E) Mature oocytes expressing Me31B::GFP (wild-type control) displaying close to spherical P body condensates. In the absence of Tral (Tral mutant), Me31B forms aberrant rod and donut-shaped P body condensates. Panel on right is a 3-D projection merge of a single donut (~2.5 μm) and rod (~1 μm) showing that they are distinct shapes (n = 20 mature oocytes). Maximum projection 5 μm.

Scale bar, 5 μm (C), 1.5 μm (D), 3 μm (E).

Götze et al., 2017; Hara et al., 2018; McCambridge et al., 2020; Monzo et al., 2006; Tritschler et al., 2007, 2008, 2009; Wang et al., 2017).

Therefore, we tested the role of Tral in P body regulation *in vivo*. Mature oocytes expressing GFP::Tral showed that Tral associates with P body condensates, albeit smaller in size than Me31B condensates (Figure 5C). Next, we asked whether Tral shows similar properties to that of Me31B, which would suggest that these two proteins are associated with the same physical state. Indeed, despite being structurally distinct from Me31B, whole FRAP and 1,6-HD experiments on Tral were consistent with our results for Me31B (Figure 5D). This supports a model in which Me31B and Tral are strongly coupled within P bodies, likely through direct interaction.

Since Me31B is essential for *Drosophila* oogenesis, we tested if Tral is required to regulate Me31B-labeled P bodies in the mature oocyte. Remarkably, in Tral mutants, P bodies have dramatically different morphologies and form rod and planar donut-shaped assemblies (Figure 5E), implying a gain of anisotropy in the underlying molecular arrangement of the condensate. The formation of apparently ordered (or partially ordered) assemblies is reminiscent of liquid-crystalline formation, as observed in the synaptonemal complex or in specific mutants of the plant protein FLOE1 (Dorone et al., 2021; Rog et al., 2017). These results suggest that, despite being structurally distinct, Tral and Me31B contribute to the organization of P bodies through synergistic interactions.

The arrested state of P bodies regulates *bcd* mRNA storage

Our data show that P bodies in the mature *Drosophila* oocyte are present in a viscous and arrested physical state. Since P bodies in the mature oocyte contain maternal mRNAs that are stored and translationally regulated over long periods, we hypothesized that the arrested physical state of P bodies could facilitate this function.

To test the hypothesis, we first developed a simple coarse-grained model in which protein and RNA will co-assemble to form condensates *in silico* (Figures 6A and S4G). In our model, protein and RNA molecules possess attractive protein-protein and protein-RNA interactions that form multicomponent condensates. Condensate stability depends on both the strength of protein-protein and protein-RNA interactions, such that over the concentration range examined, both species are necessary for condensation. In simulations where the protein-protein interaction strength is systematically weakened, we observe a loss of condensate integrity and a concomitant release of RNA into the dilute phase. These simulations predict that condensate integrity can be viewed as a proxy for RNA storage.

We first tested this prediction in live oocytes by asking if the addition of 1,6-HD could trigger the release of *bcd* mRNA, a well-established example of long-term storage, which is known to localize to P bodies in the mature oocyte (Figure 6B). Upon 1,6-HD treatment of mature oocytes, P bodies became more spherical, consistent with a loss of P body integrity and a

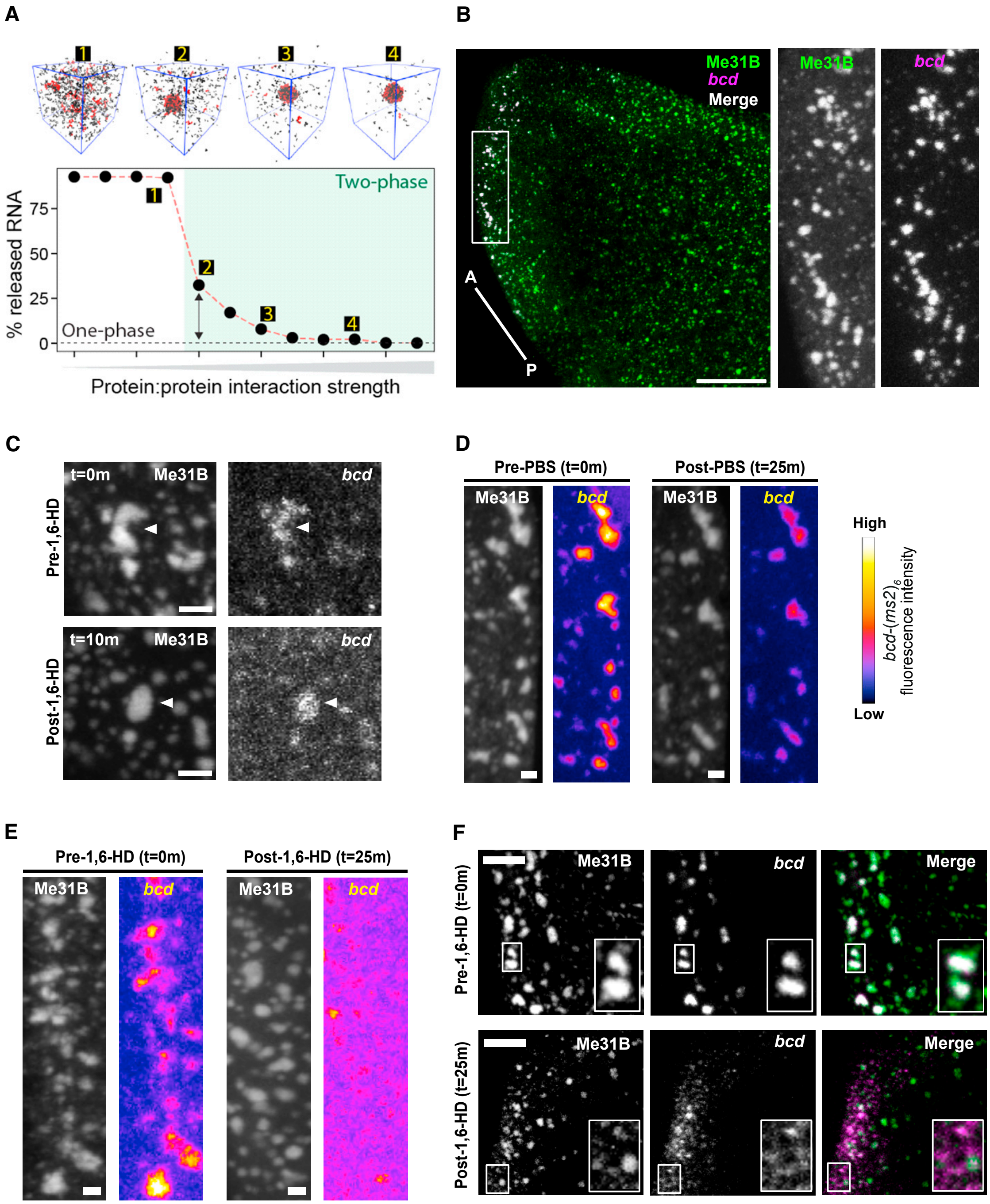

Figure 6. Altering P body physical state leads to premature loss of *bcd* mRNA

(A) Coarse-grained simulations performed with 50 RNA molecules 800 protein molecules in which condensate assembly is driven by both protein-protein and protein-RNA interactions. See also Figure S4G.

(B) Mature oocyte expressing Me31B::GFP, labeled with GFP-Booster and smFISH for *bcd* mRNA. P bodies and *bcd* mRNA co-localize at the anterior region. Inset shows a zoomed in version of *bcd* mRNA and P bodies (n = 10 mature oocytes). Maximum projection 5 μm.

(C–E) Mature oocytes expressing Me31B::GFP, *hsp83-MCP-RFP*, and *bcd-(ms2)*$_6$.

(C) The addition of 1,6-HD causes P bodies and *bcd* mRNA to initially adopt a spherical shape (t = 10 min), suggestive of a more dynamic physical state (n = 30). Maximum projection, 5 μm.

(D) The addition of PBS does not affect the co-localization of *bcd* mRNA with P bodies. Although fluorescence of *bcd* at t = 25 min decreases, this is likely due to photobleaching, the cytoplasmic distribution of *bcd* mRNA remains similar to t = 0 min (blue) (n = 35). Maximum projection 5 μm.

(E) Extended exposure to 1,6-HD results in the dispersion of *bcd* mRNAs, whereas P bodies remain condensed (t = 25 min) (n = 55). Maximum projection 5 μm.

(legend continued on next page)

CellPress
OPEN ACCESS

transition into a more dynamic state (Figure 6C). Furthermore, in line with our predictions, whereas P bodies remained relatively condensed, MS2-labeled *bcd* mRNA association with P bodies reduced dramatically post-1,6-HD treatment in comparison with phosphate buffered saline (PBS)-treated oocytes (Figures 6D, 6E, S5A, and S5B). Although we did observe a small reduction in *bcd* fluorescence in PBS-only-treated oocytes, this effect is likely due to the composition of monovalent salts present in PBS, in addition to the photobleaching effect (Figure S5A).

Since the live imaging of *bcd* mRNA with the MS2 system yields a lower signal to noise ratio than fixed samples labeled with dyes, we sought to confirm these results using smFISH coupled with immunofluorescence. In the untreated mature oocytes, *bcd* was concentrated in P bodies and we did not detect any free *bcd* mRNA that was not associated with Me31B. Alternatively, in the 1,6-HD-treated mature oocytes, more than 50% of the *bcd* mRNA particles were not associated with Me31B (Figure 6F). Together, these results suggest that the transition induced by 1,6-HD leads to the release of *bcd* mRNA, eventually resulting in P body dissolution.

Egg activation modulates P body properties and results in the release of *bcd* mRNA in the early embryo

To explore the relationship between RNA release and P body integrity in a physiological context, we examined P bodies and *bcd* mRNA at egg activation and in the early embryo. The process of egg activation is a conserved step in animal development, and previous work in *Drosophila* has shown that egg activation alone results in the widespread translation of maternal mRNAs, including *bcd* (Eichhorn et al., 2016).

To test if egg activation affects P body integrity and *bcd* mRNA association, we utilized a well-established buffer (activation buffer [AB]) to activate mature oocytes *ex vivo*. Importantly, the addition of AB mimics downstream cellular and molecular changes observed *in vivo* (Krauchunas and Wolfner, 2013; York-Andersen et al., 2015, 2020). Upon treatment with AB, both P bodies and *bcd* mRNAs underwent a rapid dispersion, consistent with a loss of P body integrity and the simultaneous release of *bcd* mRNA (Figure 7A). We also confirmed the loss of association between P bodies and *bcd* using smFISH analysis of *ex vivo* activated oocytes, which showed a dispersed distribution of *bcd* mRNA particles at the anterior, whereas Me31B was diffused (Figure 7B). This finding is consistent with data arguing that the translation of *bcd* mRNA only occurs when the mRNA is no longer inside P bodies (Eichhorn et al., 2016; Weil et al., 2012). Together, these results suggest that P bodies facilitate the storage of mRNAs, such as *bcd*, which are later released for translation, following P body dispersion at egg activation.

Following egg activation and fertilization, P body-associated proteins have been observed in early embryos; however, details of their physical properties are not known (Lin et al., 2008; Patel et al., 2016). Live imaging in the early embryo reveals P bodies that are smaller, with increased spatial mobility, and more spherical in shape than those in mature oocytes (Figures 7C and S5C–S5E). To test if embryonic P bodies exhibit an arrested state, we performed whole FRAP. In contrast to P bodies in the mature oocyte, P bodies in the early embryo exhibited rapid recovery of fluorescence and a high proportion of mobile Me31B (Figure 7D). These results collectively show that the P bodies from the early embryo are fundamentally different from those in the mature oocyte, despite being referred with the same name.

Finally, we tested if the modified P bodies in the early embryo reassociate with *bcd* mRNA, which is continuously translated in early embryogenesis. Using smFISH, we found that *bcd* mRNA particles are not associated with the re-formed P bodies, consistent with expectations for translationally active mRNAs (Figure 7E). In addition, we visualized hunchback (*hb*) mRNA, a zygotic gene required for embryo pattern formation and a downstream target of Bcd protein (Crauk and Dostatni, 2005). Interestingly, *hb* mRNA is also not associated with P bodies in the early embryo (Figure S5F). Taken together, the modified properties of P bodies in the early embryo suggest a change in P body function during the oocyte-to-embryo transition.

DISCUSSION

Over the last decade, biomolecular condensates have emerged as a key principle in cellular organization. Although changes in condensate physical properties have been examined extensively *in vitro*, the *in vivo* relevance of physical states has been explored to a lesser extent. Here, we demonstrate that a combination of intrinsic (multivalent interactions, presence of IDRs) and extrinsic (RNA, actin, and disordered proteins) factors can regulate the integrity and the arrested physical state of P bodies, both of which contribute to the storage of *bcd* mRNA in mature oocytes (Figure 7F). We also show that P bodies exhibit modified properties in the early embryo. We support a model whereby multivalent interactions, modular protein regions, and cellular factors trigger changes in the physical states of RNP condensates to facilitate differential mRNA outcomes during development.

Although dynamic, liquid-like states have been observed for many biomolecular condensates, there is a growing repertoire of functionally important and dynamically arrested condensates (Boke et al., 2016; Brangwynne et al., 2011; Hubstenberger et al., 2013; Woodruff et al., 2017). Balbiani bodies, for instance, adopt a solid-like physical state, which is thought to facilitate prolonged storage of macromolecules in dormant vertebrate oocytes (Boke et al., 2016). P bodies in *Drosophila* oocytes exhibit a physical state that allows internal mobility but prevents the exchange of proteins between the P body and the cytoplasm. Analogous states have been observed in the germline P bodies of arrested *C. elegans* oocytes (Hubstenberger et al., 2013), suggesting that the viscous properties of RNP condensates could be an evolutionarily conserved mechanism to temporally regulate mRNAs essential for normal development. Importantly, such physical states of RNP condensates may be preserved across

(F) Mature oocyte expressing Me31B::GFP, labeled with GFP-Booster and smFISH for *bcd* mRNA, before and after treatment with 1,6-HD. At t = 0 min, 98% of *bcd* mRNA particles are co-localized with P bodies. Following treatment with 1,6-HD (t = 25 min), *bcd* mRNA particles disperse and are not always co-localized with P bodies (52%) (n = 10 mature oocytes). Maximum projection 5 μm.
Scale bar, 10 μm (A), 5 μm (B and F), 1.5 μm (C–E).
See also Figure S5.

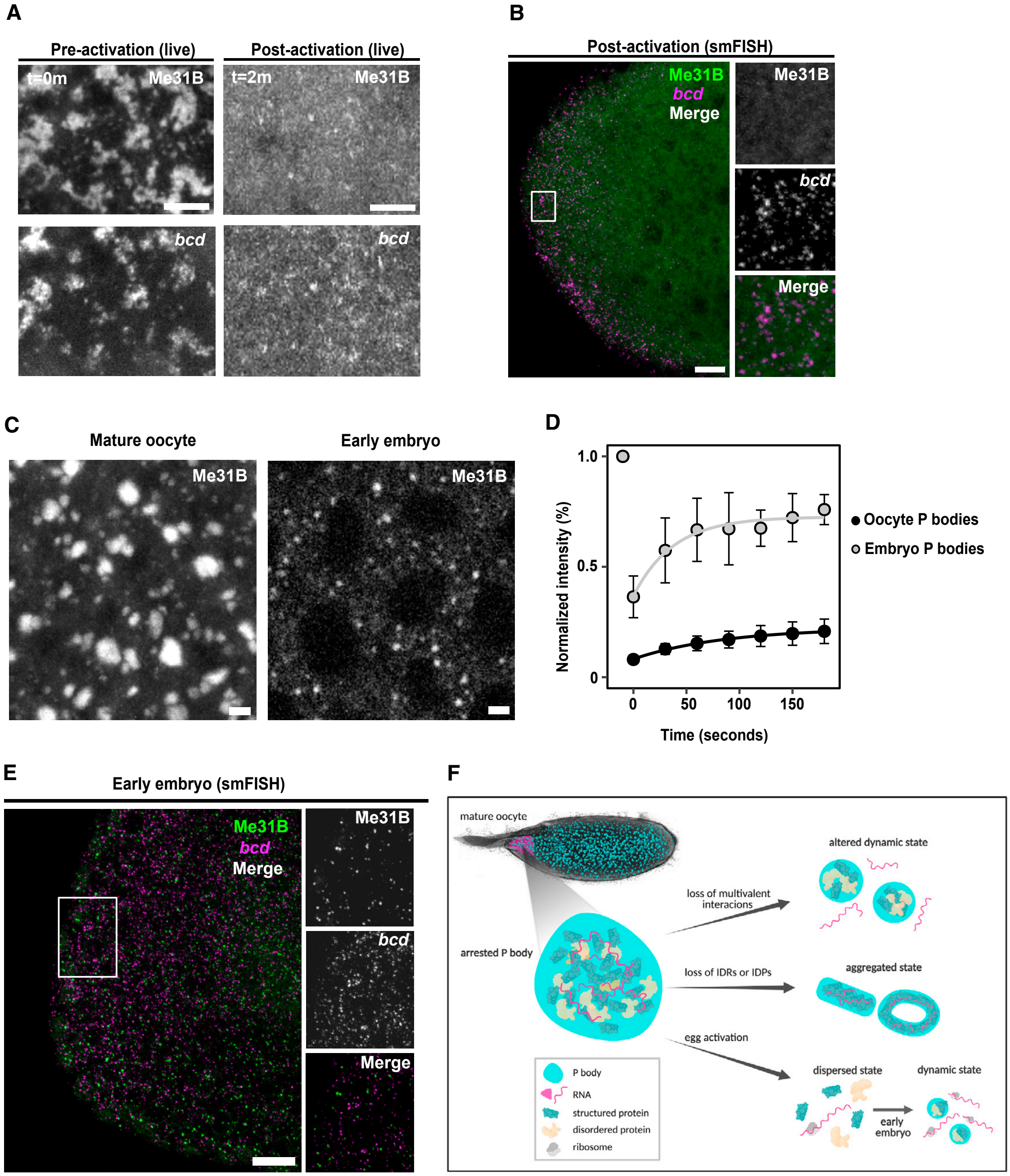

Figure 7. Egg activation modulates P body properties in the early embryo

(A–C and E) Mature oocytes expressing Me31B::GFP.

(A) The addition of activation buffer results in simultaneous dispersion of P bodies and *bcd* mRNA from condensed (t = 0 min) to diffused state (t = 2 min) (n = 0 mature oocytes). Maximum projection 5 μm.

(B) smFISH of activated oocytes stained for Me31B using a GFP-Booster and *bcd* shows diffuse P bodies and dispersed distribution of *bcd* mRNAs. Inset shows a zoomed in version of *bcd* mRNA and P body distribution (n = 10 activated oocytes). Maximum projection 5 μm.

(C) P bodies in the mature oocyte are larger than those in the early embryo (n = 50 early embryos). Max projection 3 μm.

(D) P body recovery profiles after whole FRAP of P bodies in the mature oocyte and early embryo. Mobile fraction for P bodies in the early embryo is 57% compared with 15% in the mature oocyte (n = 20 mature oocytes, n = 8 early embryos) (mean, standard deviation).

(E) smFISH of early embryos stained for Me31B using a GFP-Booster and *bcd* shows no co-localization of P bodies and *bcd* mRNAs. Inset shows a zoomed in version of *bcd* mRNA and P body distribution (n = 10 early embryos). Maximum projection 5 μm.

(F) P bodies (cyan) distributed throughout the mature *Drosophila* oocyte adopt an arrested physical state. The assembly, organization, and physical properties of P bodies are regulated by multivalent interactions between structured proteins (green) and intrinsically disordered proteins (IDP, yellow), as well as RNAs

(legend continued on next page)

other specialized cell types, such as neurons. For example, mRNAs stored and translationally repressed in neuronal RNP condensates are temporarily translated in an activity-induced manner at specific synapses, thereby influencing short-term or long-term memory (Puthanveettil, 2013; Rajasethupathy et al., 2009). Although it is not clear how this translation is regulated, models suggest that RNP granules switch between liquid-like and solid-like states to facilitate differential translation control (Bakthavachalu et al., 2018; Majumdar et al., 2012; Sudhakaran and Ramaswami, 2017).

Prior to this work, it was unclear how mRNAs could be subjected to efficient storage and differential release at distinct stages of oogenesis without obviously disrupting the integrity of P bodies (Sankaranarayanan and Weil, 2020). Previous work using cryo-immunoelectron microscopy on ultrathin frozen sections showed that maternal mRNAs are dynamically partitioned according to their translational status in mid-stage oocyte P bodies. Specifically, mRNAs that are being translated are enriched on the edge of P bodies with the cytoplasmic polyadenylation element-binding (CPEB) protein, oo18 RNA-binding protein (Orb), and ribosomes (Davidson et al., 2016; Weil et al., 2012). Alternatively, stored mRNAs that reside inside of mid-stage oocyte P bodies are repressed until later stages of development. Our data demonstrate that P body properties can be modified by attenuating multivalent hydrophobic or electrostatic interactions. Therefore, stored mRNAs could be subjected to controlled release through modulating the integrity of P bodies in response to developmental and molecular cues.

One striking observation is the influence of disordered regions in regulating the physical state of P body condensates. Conventional wisdom posits that IDRs contribute weak multivalent interactions that are essential for condensation. However, our results offer an alternative model—rather than driving assembly, IDRs may also function to modulate the physical state of condensates by counteracting the interactions driven by adhesive contact sites on folded domains. This model echoes prior work on the yeast prion protein Sup35, where the loss of N-terminal disordered regions leads to robust aggregation of the folded C-terminal domain, whereas the full-length protein rapidly assembles into dynamic condensates (Franzmann et al., 2018). The role of IDRs in the formation or regulation of RNP condensates is context dependent; the DEAD-box helicase eIF4A lacks any appreciable IDRs but serves to regulate stress granule formation in cells (Tauber et al., 2020). However, in structured proteins, which possess unusually short, disordered regions, as in Me31B, we speculate that the disordered regions may have emerged to modulate the physical states of RNP condensates.

Another key determinant that regulates biomolecular condensate formation and physical properties is multivalency. Condensates such as P bodies contain hundreds of diverse RNP components, which likely serve as a major source of multivalent interactions. Although structured and disordered RNA-binding proteins have been investigated previously, how they influence the overall property of condensates *in vivo* is unclear. Using Me31B and Tral, our results indicate that structurally distinct proteins synergistically interact to regulate P bodies during *Drosophila* oogenesis. These data also agree with observations reported for Tral and Me31B orthologs in arrested *C. elegans* oocytes (Hubstenberger et al., 2013), suggesting that the underlying physical interactions between RNP components may be evolutionarily conserved. Overall, our *in vivo* and *in vitro* data indicate that IDRs act as lubricants to regulate the overall physical state and organization of P bodies through interactions with structured proteins.

The oocyte-to-embryo transition is accompanied by large-scale changes in the cytoplasm, including the translation of stored mRNAs and widespread post-translational modifications (PTMs) (Eichhorn et al., 2016; Hara et al., 2018). In fact, Me31B is known to be phosphorylated and ubiquitinated in the early embryo (Hara et al., 2018; Zavortink et al., 2020). Such modifications have been shown to alter the physical properties of RNP condensates *in vitro* (Hofweber and Dormann, 2019; Owen and Shewmaker, 2019; Schisa and Elaswad, 2021). Our data from the early embryo suggest that PTMs may be influencing the modification of P body physical state and function post-egg activation.

One open question relates to the molecular differences between P bodies in the oocyte and early embryo. *In vitro* studies have shown that RNA can modulate the properties of condensates, including their size and dynamics (Garcia-Jove Navarro et al., 2019; Roden and Gladfelter, 2021). Our *in vivo* data showing smaller-sized oocyte P bodies after RNase A treatment and the absence of actively translating mRNAs in embryonic P bodies led us to speculate that a decrease in the abundance, or absence, of RNAs is likely contributing to changes in P body size and dynamics.

Finally, biochemical and molecular evidence suggest that P body proteins, including Me31B and Tral, change primary function from translational repression to degradation in the early embryo (Wang et al., 2017). We suspect the changes that accompany the dissolution of P bodies at egg activation, and their re-condensation in the embryo, reflect developmentally required transitions in mRNA regulation (Hubstenberger et al., 2013; Kato and Nakamura, 2012; Wang et al., 2017). In line with this model, we propose a general framework whereby developmental cues coordinate molecular interactions and large-scale cytoplasmic modifications to regulate mRNAs via adaptable RNP physical states.

Limitations of the study

Our work shows that the arrested physical state of P bodies facilitates the storage of *bcd* mRNA in the mature oocyte. Whether such a state also facilitates mRNA storage during earlier stages of oogenesis, or in other cells, remains to be determined. Additionally, more mRNAs need to be tested to expand the relevance of the arrested state. Another limitation is the use of 1,6-HD to study P body physical state and *bcd* regulation. Although 1,6-HD has been commonly used to attenuate hydrophobic

(magenta). The loss of these interactions alters the physical state of P bodies. At egg activation, P bodies disperse and release stored RNA for translation. In early embryogenesis, P bodies re-condense but are more dynamic and do not co-localize with RNAs. Created with BioRender.com.
Scale bar, 2 μm (A and C), 5 μm (B, D and E).
See also Figure S4.

interactions, it is not a naturally occurring cellular factor. Therefore, identifying cellular components that interfere with P body properties and studying the consequences of premature release of stored mRNAs is worthy of further investigation. Finally, we note that although 1,6-HD treatment results in the release of *bcd* from P bodies, we did not detect *bcd* translation (data not shown). This is likely due either to the requirement of a translational activator (or loss of a translational repressor) to initiate translation at egg activation or to the ability of 1,6-HD to impair kinase and phosphatase activities, which are thought to regulate translation of mRNAs (Düster et al., 2021). Whether or not the release of mRNA from P bodies alone is sufficient for translation continues to be an important area of investigation in the future.

STAR★METHODS

Detailed methods are provided in the online version of this paper and include the following:

- KEY RESOURCES TABLE
- RESOURCE AVAILABILITY
  - Lead contact
  - Materials availability
  - Data and code availability
- EXPERIMENTAL MODEL AND SUBJECT DETAILS
  - Drosophila stocks
- METHOD DETAILS
  - Oocyte sample preparation
  - Live imaging
  - Pharmacological treatments
  - Protein purification
  - *In vitro* condensation assay
  - Optical tweezer experiments
  - Fluorescence recovery after photobleaching
  - Single molecule fluorescence *in situ* hybridization
  - *In situ* hybridization chain reaction V3.0
- QUANTIFICATION AND STATISTICAL ANALYSIS
  - Optical tweezer experiments
  - Fluorescence recovery after photobleaching
  - Fluorescence intensity measurements
  - Aspect ratio, circularity and particle area
  - Apparent viscosity estimation
  - Particle displacement analysis
  - All-atom simulations
  - Bioinformatics
  - Coarse-grained simulations
- ADDITIONAL RESOURCES

SUPPLEMENTAL INFORMATION

Supplemental information can be found online at https://doi.org/10.1016/j.devcel.2021.09.021.

ACKNOWLEDGMENTS

We thank the Bloomington Drosophila Stock Center, Ilan, Davis, Liz Gavis, James Wilhelm, and William Chia for fly stocks. We are grateful to Paul Conduit for experimental advice; Titus Franzmann for advice on FRAP analysis; the Zoology Imaging Facility for assistance and support with microscopy; and funding from the University of Cambridge ISSF (097814) and the Wellcome Trust (200734/Z/16/Z) (to T.T.W.), INLAKS, the Cambridge Trust scholarship (to M.S.), the BBSRC DTP scholarship (to E.L.W.), William H. Danforth Plant Science Fellowship (to R.J.E), and the Technische Universität Dresden and the European Research Council (PhaseAge, No. 725836) (to S.A).

AUTHOR CONTRIBUTIONS

Conceptualization, M.S. and T.T.W.; methodology, M.S., A.S.H., and T.T.W.; investigation, majority, M.S.; investigation, simulations and *in silico* modeling, R.J.E. and A.S.H.; investigation, protein purification, I.R.E.A.T.; investigation, optical tweezer, M.J.; investigation, smFISH and tracking, E.L.W.; formal analysis, M.W.; writing – original draft, M.S. and T.T.W.; writing – review & editing, M.S., A.S.H., S.A., and T.T.W.; funding acquisition, M.W., A.S.H., S.A., and T.T.W.; supervision, A.S.H., S.A., and T.T.W.

DECLARATIONS OF INTERESTS

S.A. is an advisor on the scientific advisory board of Dew point Therapeutics. A.S.H. is a scientific consultant with Dewpoint Therapeutics.

Received: March 21, 2021
Revised: July 22, 2021
Accepted: September 23, 2021
Published: October 15, 2021

REFERENCES

Alshareedah, I., Thurston, G.M., and Banerjee, P.R. (2021). Quantifying viscosity and surface tension of multicomponent protein-nucleic acid condensates. Biophys. J. *120*, 1161–1169.

Andrei, M.A., Ingelfinger, D., Heintzmann, R., Achsel, T., Rivera-Pomar, R., and Lührmann, R. (2005). A role for eIF4E and eIF4E-transporter in targeting mRNPs to mammalian processing bodies. RNA *11*, 717–727.

Bakthavachalu, B., Huelsmeier, J., Sudhakaran, I.P., Hillebrand, J., Singh, A., Petrauskas, A., Thiagarajan, D., Sankaranarayanan, M., Mizoue, L., Anderson, E.N., et al. (2018). RNP-granule assembly via ataxin-2 disordered domains is required for long-term memory and neurodegeneration. Neuron *98*, 754–766.e4.

Banani, S.F., Lee, H.O., Hyman, A.A., and Rosen, M.K. (2017). Biomolecular condensates: organizers of cellular biochemistry. Nat. Rev. Mol. Cell Biol. *18*, 285–298.

Boeynaems, S., Alberti, S., Fawzi, N.L., Mittag, T., Polymenidou, M., Rousseau, F., Schymkowitz, J., Shorter, J., Wolozin, B., Van Den Bosch, L., et al. (2018). Protein phase separation: a new phase in cell biology. Trends Cell Biol. *28*, 420–435.

Boeynaems, S., Holehouse, A.S., Weinhardt, V., Kovacs, D., Van Lindt, J., Larabell, C., Van Den Bosch, L., Das, R., Tompa, P.S., Pappu, R.V., and Gitler, A.D. (2019). Spontaneous driving forces give rise to protein−RNA condensates with coexisting phases and complex material properties. Proc. Natl. Acad. Sci. USA *116*, 7889–7898.

Boke, E., Ruer, M., Wühr, M., Coughlin, M., Lemaitre, R., Gygi, S.P., Alberti, S., Drechsel, D., Hyman, A.A., and Mitchison, T.J. (2016). Amyloid-like self-assembly of a cellular compartment. Cell *166*, 637–650.

Bouveret, E., Rigaut, G., Shevchenko, A., Wilm, M., and Séraphin, B. (2000). A Sm-like protein complex that participates in mRNA degradation. EMBO J. *19*, 1661–1671.

Brangwynne, C.P., Eckmann, C.R., Courson, D.S., Rybarska, A., Hoege, C., Gharakhani, J., Jülicher, F., and Hyman, A.A. (2009). Germline P granules are liquid droplets that localize by controlled dissolution/condensation. Science *324*, 1729–1732.

Brangwynne, C.P., Mitchison, T.J., and Hyman, A.A. (2011). Active liquid-like behavior of nucleoli determines their size and shape in Xenopus laevis oocytes. Proc. Natl. Acad. Sci. USA *108*, 4334–4339.

Brangwynne, C.P., Tompa, P., and Pappu, R.V. (2015). Polymer physics of intracellular phase transitions. Nature Phys. *11*, 899–904.

Buchan, J.R., and Parker, R. (2009). Eukaryotic stress granules: the ins and outs of translation. Mol. Cell *36*, 932–941.

CellPress
OPEN ACCESS

Buchan, J.R. (2014). mRNP granules. Assembly, function, and connections with disease. RNA Biol. *11*, 1019–1030.

Buszczak, M., Paterno, S., Lighthouse, D., Bachman, J., Planck, J., Owen, S., Skora, A.D., Nystul, T.G., Ohlstein, B., Allen, A., et al. (2007). The carnegie protein trap library: a versatile tool for Drosophila developmental studies. Genetics *175*, 1505–1531.

Choi, H.M.T., Schwarzkopf, M., Fornace, M.E., Acharya, A., Artavanis, G., Stegmaier, J., Cunha, A., and Pierce, N.A. (2018). Third-generation in situ hybridization chain reaction: multiplexed, quantitative, sensitive, versatile, robust. Development *145*, dev165753.

Crauk, O., and Dostatni, N. (2005). Bicoid determines sharp and precise target gene expression in the Drosophila embryo. Curr. Biol. *15*, 1888–1898.

Cubuk, J., Alston, J.J., Incicco, J.J., Singh, S., Stuchell-Brereton, M.D., Ward, M.D., Zimmerman, M.I., Vithani, N., Griffith, D., Wagoner, J.A., et al. (2021). The SARS-CoV-2 nucleocapsid protein is dynamic, disordered, and phase separates with RNA. Nat. Commun. *12*, 1936.

Davidson, A., Parton, R.M., Rabouille, C., Weil, T.T., and Davis, I. (2016). Localized translation of gurken/TGF-α mRNA during axis specification is controlled by access to orb/CPEB on processing bodies. Cell Rep. *14*, 2451–2462.

Derrick, C.J., York-Andersen, A.H., and Weil, T.T. (2016). Imaging calcium in Drosophila at egg activation. J. Vis. Exp. *114*, e54311.

Dorone, Y., Boeynaems, S., Flores, E., Jin, B., Hateley, S., Bossi, F., Lazarus, E., Pennington, J.G., Michiels, E., De Decker, M., et al. (2021). A prion-like protein regulator of seed germination undergoes hydration-dependent phase separation. Cell *184*, 4284–4298.e27.

Düster, R., Kaltheuner, I.H., Schmitz, M., and Geyer, M. (2021). 1,6-hexanediol, commonly used to dissolve liquid-liquid phase separated condensates, directly impairs kinase and phosphatase activities. J. Biol. Chem. *296*, 100260.

Dzuricky, M., Rogers, B.A., Shahid, A., Cremer, P.S., and Chilkoti, A. (2020). De novo engineering of intracellular condensates using artificial disordered proteins. Nat. Chem. *12*, 814–825.

Eichhorn, S.W., Subtelny, A.O., Kronja, I., Kwasnieski, J.C., Orr-Weaver, T.L., and Bartel, D.P. (2016). mRNA poly(A)-tail changes specified by deadenylation broadly reshape translation in Drosophila oocytes and early embryos. eLife *5*, 1–24.

Elbaum-Garfinkle, S., Kim, Y., Szczepaniak, K., Chen, C.C.-H., Eckmann, C.R., Myong, S., and Brangwynne, C.P. (2015). The disordered P granule protein LAF-1 drives phase separation into droplets with tunable viscosity and dynamics. Proc. Natl. Acad. Sci. U S A *112*, 7189–7194.

Emenecker, R.J., Griffith, D., and Holehouse, A.S. (2021). metapredict: a fast, accurate, and easy-to-use predictor of consensus disorder and structure. bioRxiv. https://doi.org/10.1101/2021.05.30.446349.

Eulalio, A., Behm-Ansmant, I., Schweizer, D., and Izaurralde, E. (2007). P-body formation is a consequence, not the cause, of RNA-mediated gene silencing. Mol. Cell. Biol. *27*, 3970–3981.

Fei, J., Jadaliha, M., Harmon, T.S., Li, I.T.S., Hua, B., Hao, Q., Holehouse, A.S., Reyer, M., Sun, Q., Freier, S.M., et al. (2017). Quantitative analysis of multilayer organization of proteins and RNA in nuclear speckles at super resolution. J. Cell Sci. *130*, 4180–4192.

Feric, M., Vaidya, N., Harmon, T.S., Mitrea, D.M., Zhu, L., Richardson, T.M., Kriwacki, R.W., Pappu, R.V., and Brangwynne, C.P. (2016). Coexisting liquid phases underlie nucleolar subcompartments. Cell *165*, 1686–1697.

Fleming, P.J., and Fleming, K.G. (2018). HullRad: fast calculations of folded and disordered protein and nucleic acid hydrodynamic properties. Biophys. J. *114*, 856–869.

Franzmann, T.M., Jahnel, M., Pozniakovsky, A., Mahamid, J., Holehouse, A.S., Nüske, E., Richter, D., Baumeister, W., Grill, S.W., Pappu, R.V., et al. (2018). Phase separation of a yeast prion protein promotes cellular fitness. Science *359*, eaao5654.

Garcia-Jove Navarro, M., Kashida, S., Chouaib, R., Souquere, S., Pierron, G., Weil, D., and Gueroui, Z. (2019). RNA is a critical element for the sizing and the composition of phase-separated RNA–protein condensates. Nat. Commun. *10*, 3230.

Götze, M., Dufourt, J., Ihling, C., Rammelt, C., Pierson, S., Sambrani, N., Temme, C., Sinz, A., Simonelig, M., and Wahle, E. (2017). Translational repression of the Drosophila nanos mRNA involves the RNA helicase Belle and RNA coating by Me31B and Trailer hitch. RNA *23*, 1552–1568.

Hara, M., Lourido, S., Petrova, B., Lou, H.J., Von Stetina, J.R., Kashevsky, H., Turk, B.E., and Orr-Weaver, T.L. (2018). Identification of PNG kinase substrates uncovers interactions with the translational repressor TRAL in the oocyte-to-embryo transition. eLife *7*, 1–19.

Hofweber, M., and Dormann, D. (2019). Friend or foe-Post-translational modifications as regulators of phase separation and RNP granule dynamics. J. Biol. Chem. *294*, 7137–7150.

Holehouse, A.S., Das, R.K., Ahad, J.N., Richardson, M.O.G., and Pappu, R.V. (2017). CIDER: resources to analyze sequence-ensemble relationships of intrinsically disordered proteins. Biophys. J. *112*, 16–21.

Holehouse, A.S., Garai, K., Lyle, N., Vitalis, A., and Pappu, R.V. (2015). Quantitative assessments of the distinct contributions of polypeptide backbone amides versus side chain groups to chain expansion via chemical denaturation. J. Am. Chem. Soc. *137*, 2984–2995.

Hondele, M., Sachdev, R., Heinrich, S., Wang, J., Vallotton, P., Fontoura, B.M.A., and Weis, K. (2019). DEAD-box ATPases are global regulators of phase-separated organelles. Nature *573*, 144–148.

Hubstenberger, A., Courel, M., Bénard, M., Souquere, S., Ernoult-Lange, M., Chouaib, R., Yi, Z., Morlot, J.B., Munier, A., Fradet, M., et al. (2017). P-body purification reveals the condensation of repressed mRNA regulons. Mol. Cell *68*, 144–157.e5.

Hubstenberger, A., Noble, S.L., Cameron, C., and Evans, T.C. (2013). Translation repressors, an RNA helicase, and developmental cues control RNP phase transitions during early development. Dev. Cell *27*, 161–173.

Hyman, A.A., Weber, C.A., and Julicher, F. (2014). Liquid-liquid phase separation in biology. Annu. Rev. Cell Dev. Biol. *30*, 39–58.

Jahnel, M., Behrndt, M., Jannasch, A., Schäffer, E., and Grill, S.W. (2011). Measuring the complete force field of an optical trap. Opt. Lett. *36*, 1260–1262.

Jung, H., Gkogkas, C.G., Sonenberg, N., and Holt, C.E. (2014). Remote control of gene function by local translation. Cell *157*, 26–40.

Kabsch, W., and Sander, C. (1983). Dictionary of protein secondary structure: pattern recognition of hydrogen-bonded and geometrical features. Biopolymers *22*, 2577–2637.

Kaneuchi, T., Sartain, C.V., Takeo, S., Horner, V.L., Buehner, N.A., Aigaki, T., and Wolfner, M.F. (2015). Calcium waves occur as Drosophila oocytes activate. Proc. Natl. Acad. Sci. USA *112*, 791–796.

Kato, M., Han, T.W., Xie, S., Shi, K., Du, X., Wu, L.C., Mirzaei, H., Goldsmith, E.J., Longgood, J., Pei, J., et al. (2012). Cell-free formation of RNA granules: low complexity sequence domains form dynamic fibers within hydrogels. Cell *149*, 753–767.

Kato, Y., and Nakamura, A. (2012). Roles of cytoplasmic RNP granules in intracellular RNA localization and translational control in the Drosophila oocyte. Dev. Growth Differ. *54*, 19–31.

Kedersha, N., Stoecklin, G., Ayodele, M., Yacono, P., Lykke-Andersen, J., Fritzler, M.J., Scheuner, D., Kaufman, R.J., Golan, D.E., and Anderson, P. (2005). Stress granules and processing bodies are dynamically linked sites of mRNP remodeling. J. Cell Biol. *169*, 871–884.

Kloc, M., and Etkin, L.D. (2005). RNA localization mechanisms in oocytes. J. Cell Sci. *118*, 269–282.

Krauchunas, A.R., and Wolfner, M.F. (2013). Molecular changes during egg activation. Curr. Top. Dev. Biol. *102*, 267–292.

Kroschwald, S., Maharana, S., Mateju, D., Malinovska, L., Nüske, E., Poser, I., Richter, D., and Alberti, S. (2015). Promiscuous interactions and protein disaggregases determine the material state of stress-inducible RNP granules. eLife *4*, e06807.

Kroschwald, S., Munder, M.C., Maharana, S., Franzmann, T.M., Richter, D., Ruer, M., Hyman, A.A., and Alberti, S. (2018). Different material states of Pub1 condensates define distinct modes of stress adaptation and recovery. Cell Rep. *23*, 3327–3339.

Lasko, P. (2012). mRNA localization and translational control in Drosophila oogenesis. Cold Spring Harb. Perspect. Biol. *4*, 1–15.

Lemaitre, R.P., Bogdanova, A., Borgonovo, B., Woodruff, J.B., and Drechsel, D.N. (2019). FlexiBAC: a versatile, open-source baculovirus vector system for protein expression, secretion, and proteolytic processing. BMC Biotechnol *19*, 20.

Li, P., Banjade, S., Cheng, H.C., Kim, S., Chen, B., Guo, L., Llaguno, M., Hollingsworth, J.V., King, D.S., Banani, S.F., et al. (2012). Phase transitions in the assembly of multivalent signalling proteins. Nature *483*, 336–340.

Lin, M.D., Jiao, X., Grima, D., Newbury, S.F., Kiledjian, M., and Chou, T.B. (2008). Drosophila processing bodies in oogenesis. Dev. Biol. *322*, 276–288.

Luo, Y., Na, Z., and Slavoff, S.A. (2018). P-bodies: composition, properties, and functions. Biochemistry *57*, 2424–2431.

Lyon, A.S., Peeples, W.B., and Rosen, M.K. (2021). A framework for understanding the functions of biomolecular condensates across scales. Nat. Rev. Mol. Cell Biol. *22*, 215–235.

Majumdar, A., Cesario, W.C., White-Grindley, E., Jiang, H., Ren, F., Khan, M.R., Li, L., Choi, E.-M., Kannan, K., Guo, F., et al. (2012). Critical role of amyloid-like oligomers of Drosophila Orb2 in the persistence of memory. Cell *148*, 515–529.

Mao, A.H., and Pappu, R.V. (2012). Crystal lattice properties fully determine short-range interaction parameters for alkali and halide ions. J. Chem. Phys. *137*, 064104.

Martin, E.W., Holehouse, A.S., Peran, I., Farag, M., Incicco, J.J., Bremer, A., Grace, C.R., Soranno, A., Pappu, R.V., and Mittag, T. (2020). Valence and patterning of aromatic residues determine the phase behavior of prion-like domains. Science *367*, 694–699.

Martin, E.W., and Mittag, T. (2018). Relationship of sequence and phase separation in protein low-complexity regions. Biochemistry *57*, 2478–2487.

McCambridge, A., Solanki, D., Olchawa, N., Govani, N., Trinidad, J.C., and Gao, M. (2020). Comparative proteomics reveal Me31B's interactome dynamics, expression regulation, and assembly mechanism into germ granules during Drosophila germline development. Sci. Rep. *10*, 564.

McGibbon, R.T., Beauchamp, K.A., Harrigan, M.P., Klein, C., Swails, J.M., Hernández, C.X., Schwantes, C.R., Wang, L.P., Lane, T.J., and Pande, V.S. (2015). MDTraj: a modern open library for the analysis of molecular dynamics trajectories. Biophys. J. *109*, 1528–1532.

Medioni, C., Mowry, K., and Besse, F. (2012). Principles and roles of mRNA localization in animal development. Development *139*, 3263–3276.

Mitrea, D.M., Cika, J.A., Guy, C.S., Ban, D., Banerjee, P.R., Stanley, C.B., Nourse, A., Deniz, A.A., and Kriwacki, R.W. (2016). Nucleophosmin integrates within the nucleolus via multi-modal interactions with proteins displaying R-rich linear motifs and rRNA. eLife *5*, e13571.

Monzo, K., Papoulas, O., Cantin, G.T., Wang, Y., Yates, J.R., and Sisson, J.C. (2006). Fragile X mental retardation protein controls trailer hitch expression and cleavage furrow formation in Drosophila embryos. Proc. Natl. Acad. Sci. USA *103*, 18160–18165.

Morin, X., Daneman, R., Zavortink, M., and Chia, W. (2001). A protein trap strategy to detect GFP-tagged proteins expressed from their endogenous loci in Drosophila. Proc. Natl. Acad. Sci. USA *98*, 15050–15055.

Murthy, A.C., Dignon, G.L., Kan, Y., Zerze, G.H., Parekh, S.H., Mittal, J., and Fawzi, N.L. (2019). Molecular interactions underlying liquid−liquid phase separation of the FUS low-complexity domain. Nat. Struct. Mol. Biol. *26*, 637–648.

Nakamura, A., Amikura, R., Hanyu, K., and Kobayashi, S. (2001). Me31B silences translation of oocyte-localizing RNAs through the formation of cytoplasmic RNP complex during Drosophila oogenesis. Development *128*, 3233–3242.

Nakamura, A., Sato, K., and Hanyu-Nakamura, K. (2004). Drosophila cup is an eIF4E binding protein that associates with Bruno and regulates oskar mRNA translation in oogenesis. Dev. Cell *6*, 69–78.

Necci, M., Piovesan, D., Dosztányi, Z., and Tosatto, S.C.E. (2017). MobiDB-lite: fast and highly specific consensus prediction of intrinsic disorder in proteins. Bioinformatics *33*, 1402–1404.

Newcombe, E.A., Ruff, K.M., Sethi, A., Ormsby, A.R., Ramdzan, Y.M., Fox, A., Purcell, A.W., Gooley, P.R., Pappu, R.V., and Hatters, D.M. (2018). Tadpole-like conformations of huntingtin exon 1 are characterized by conformational heterogeneity that persists regardless of polyglutamine length. J. Mol. Biol. *430*, 1442–1458.

Nott, T.J., Petsalaki, E., Farber, P., Jervis, D., Fussner, E., Plochowietz, A., Craggs, T.D., Bazett-Jones, D.P., Pawson, T., Forman-Kay, J.D., et al. (2015). Phase transition of a disordered nuage protein generates environmentally responsive membraneless organelles. Mol. Cell *57*, 936–947.

Owen, I., and Shewmaker, F. (2019). The role of post-translational modifications in the phase transitions of intrinsically disordered proteins. Int. J. Mol. Sci. *20*, 5501.

Pak, C.W., Kosno, M., Holehouse, A.S., Padrick, S.B., Mittal, A., Ali, R., Yunus, A.A., Liu, D.R., Pappu, R.V., and Rosen, M.K. (2016). Sequence determinants of intracellular phase separation by complex coacervation of a disordered protein. Mol. Cell *63*, 72–85.

Parker, R., and Sheth, U. (2007). P bodies and the control of mRNA translation and degradation. Mol. Cell *25*, 635–646.

Patel, A., Lee, H.O., Jawerth, L., Maharana, S., Jahnel, M., Hein, M.Y., Stoynov, S., Mahamid, J., Saha, S., Franzmann, T.M., et al. (2015). A liquid-to-solid phase transition of the ALS protein FUS accelerated by disease mutation. Cell *162*, 1066–1077.

Patel, P.H., Barbee, S.A., and Blankenship, J.T. (2016). GW-bodies and P-bodies constitute two separate pools of sequestered non-translating RNAs. PLoS One *11*, e0150291.

Patel, S.S., Belmont, B.J., Sante, J.M., and Rexach, M.F. (2007). Natively unfolded nucleoporins gate protein diffusion across the nuclear pore complex. Cell *129*, 83–96.

Piovesan, D., Necci, M., Escobedo, N., Monzon, A.M., Hatos, A., Mičetić, I., Quaglia, F., Paladin, L., Ramasamy, P., Dosztányi, Z., et al. (2021). MobiDB: intrinsically disordered proteins in 2021. Nucleic Acids Res. *49*, D361–D367.

Puthanveettil, S.V. (2013). RNA transport and long-term memory storage. RNA Biol. *10*, 1765–1770.

Rajasethupathy, P., Fiumara, F., Sheridan, R., Betel, D., Puthanveettil, S.V., Russo, J.J., Sander, C., Tuschl, T., and Kandel, E. (2009). Characterization of small RNAs in Aplysia reveals a role for miR-124 in constraining synaptic plasticity through CREB. Neuron *63*, 803–817.

Riback, J.A., Katanski, C.D., Kear-Scott, J.L., Pilipenko, E.V., Rojek, A.E., Sosnick, T.R., and Drummond, D.A. (2017). Stress-triggered phase separation is an adaptive, evolutionarily tuned response. Cell *168*, 1028–1040.e19.

Ribbeck, K., and Görlich, D. (2002). The permeability barrier of nuclear pore complexes appears to operate via hydrophobic exclusion. EMBO J. *21*, 2664–2671.

Roden, C., and Gladfelter, A.S. (2021). RNA contributions to the form and function of biomolecular condensates. Nat. Rev. Mol. Cell Biol. *22*, 183–195.

Rog, O., Köhler, S., and Dernburg, A.F. (2017). The synaptonemal complex has liquid crystalline properties and spatially regulates meiotic recombination factors. eLife *6*, e21455.

Sankaranarayanan, M., and Weil, T.T. (2020). Granule regulation by phase separation during Drosophila oogenesis. Emerg. Top. Life Sci. *4*, 343–352.

Schisa, J.A., and Elaswad, M.T. (2021). An emerging role for post-translational modifications in regulating RNP condensates in the germ line. Front. Mol. Biosci. *8*, 658020.

Schneider, C.A., Rasband, W.S., and Eliceiri, K.W. (2012). NIH Image to ImageJ: 25 years of image analysis. Nat. Methods *9*, 671–675.

Sheth, U., and Parker, R. (2003). Decapping and decay of messenger RNA occur in cytoplasmic processing bodies. Science *300*, 805–808.

Shin, Y., and Brangwynne, C.P. (2017). Liquid phase condensation in cell physiology and disease. Science *357*, eaaf4382.

Sudhakaran, I.P., and Ramaswami, M. (2017). Long-term memory consolidation : the role of RNA- binding proteins with prion-like domains. RNA Biol. *14*, 568–586.

Tadros, W., and Lipshitz, H.D. (2009). The maternal-to-zygotic transition : a play in two acts. Development *136*, 3033–3042.

Tauber, D., Tauber, G., Khong, A., Van Treeck, B., Pelletier, J., and Parker, R. (2020). Modulation of RNA condensation by the DEAD-box protein eIF4A. Cell *180*, 411–426.e16.

Tinevez, J.Y., Perry, N., Schindelin, J., Hoopes, G.M., Reynolds, G.D., Laplantine, E., Bednarek, S.Y., Shorte, S.L., and Eliceiri, K.W. (2017). TrackMate: an open and extensible platform for single-particle tracking. Methods *115*, 80–90.

Tritschler, F., Braun, J.E., Eulalio, A., Truffault, V., Izaurralde, E., and Weichenrieder, O. (2009). Structural basis for the mutually exclusive anchoring of P Body components EDC3 and Tral to the DEAD box protein DDX6/Me31B. Mol. Cell *33*, 661–668.

Tritschler, F., Eulalio, A., Helms, S., Schmidt, S., Coles, M., Weichenrieder, O., Izaurralde, E., and Truffault, V. (2008). Similar modes of interaction enable trailer hitch and EDC3 to associate with DCP1 and Me31B in distinct protein complexes. Mol. Cell. Biol. *28*, 6695–6708.

Tritschler, F., Eulalio, A., Truffault, V., Hartmann, M.D., Helms, S., Schmidt, S., Coles, M., Izaurralde, E., and Weichenrieder, O. (2007). A divergent Sm Fold in EDC3 proteins mediates DCP1 binding and P-body targeting. Mol. Cell. Biol. *27*, 8600–8611.

Trovisco, V., Belaya, K., Nashchekin, D., Irion, U., Sirinakis, G., Butler, R., Lee, J.J., Gavis, E.R., and St Johnston, D. (2016). bicoid mRNA localises to the Drosophila oocyte anterior by random Dynein-mediated transport and anchoring. eLife *5*, e17537.

Vitalis, A., and Pappu, R.V. (2009). ABSINTH: a new continuum solvation model for simulations of polypeptides in aqueous solutions. J. Comput. Chem. *30*, 673–699.

Wang, J.T., Smith, J., Chen, B.C., Schmidt, H., Rasoloson, D., Paix, A., Lambrus, B.G., Calidas, D., Betzig, E., and Seydoux, G. (2014). Regulation of RNA granule dynamics by phosphorylation of serine-rich, intrinsically disordered proteins in C. elegans. eLife *3*, e04591.

Wang, M., Ly, M., Lugowski, A., Laver, J.D., Lipshitz, H.D., Smibert, C.A., and Rissland, O.S. (2017). ME31B globally represses maternal mRNAs by two distinct mechanisms during the Drosophila maternal-to-zygotic transition. eLife *6*, 1–22.

Waterhouse, A., Bertoni, M., Bienert, S., Studer, G., Tauriello, G., Gumienny, R., Heer, F.T., de Beer, T.A.P., Rempfer, C., Bordoli, L., et al. (2018). SWISS-MODEL: homology modelling of protein structures and complexes. Nucleic Acids Res. *46*, W296–W303.

Weber, S.C. (2017). Sequence-encoded material properties dictate the structure and function of nuclear bodies. Curr. Opin. Cell Biol. *46*, 62–71.

Weber, S.C., and Brangwynne, C.P. (2012). Getting RNA and protein in phase. Cell *149*, 1188–1191.

Weil, T.T., Forrest, K.M., and Gavis, E.R. (2006). Localization of bicoid mRNA in late oocytes is maintained by continual active transport. Dev. Cell *11*, 251–262.

Weil, T.T., Parton, R., Davis, I., and Gavis, E.R. (2008). Changes in bicoid mRNA anchoring highlight conserved mechanisms during the oocyte-to-embryo transition. Curr. Biol. *18*, 1055–1061.

Weil, T.T., Parton, R.M., and Davis, I. (2012b). Preparing individual Drosophila egg chambers for live imaging. J. Vis. Exp. *60*, e3679.

Weil, T.T., Parton, R.M., Herpers, B., Soetaert, J., Veenendaal, T., Xanthakis, D., Dobbie, I.M., Halstead, J.M., Hayashi, R., Rabouille, C., and Davis, I. (2012). Drosophila patterning is established by differential association of mRNAs with P bodies. Nat. Cell Biol. *14*, 1305–1313.

Wheeler, J.R., Matheny, T., Jain, S., Abrisch, R., and Parker, R. (2016). Distinct stages in stress granule assembly and disassembly. eLife *5*, e18413.

Wilhelm, J.E., Buszczak, M., and Sayles, S. (2005). Efficient protein trafficking requires trailer hitch, a component of a ribonucleoprotein complex localized to the ER in Drosophila. Dev. Cell *9*, 675–685.

Woodruff, J.B., Ferreira Gomes, B., Widlund, P.O., Mahamid, J., Honigmann, A., and Hyman, A.A. (2017). The centrosome is a selective condensate that nucleates microtubules by concentrating tubulin. Cell *169*, 1066–1077.e10.

York-Andersen, A.H., Hu, Q., Wood, B.W., Wolfner, M.F., and Weil, T.T. (2020). A calcium-mediated actin redistribution at egg activation in Drosophila. Mol. Reprod. Dev. *87*, 293–304.

York-Andersen, A.H., Parton, R.M., Bi, C.J., Bromley, C.L., Davis, I., and Weil, T.T. (2015). A single and rapid calcium wave at egg activation in Drosophila. Biol. Open *4*, 553–560.

Zavortink, M., Rutt, L.N., Dzitoyeva, S., Henriksen, J.C., Barrington, C., Bilodeau, D.Y., Wang, M., Chen, X.X.L., and Rissland, O.S. (2020). The E2 Marie Kondo and the CTLH E3 ligase clear deposited RNA binding proteins during the maternal-to-zygotic transition. eLife *9*, e53889.

Zhang, H., Elbaum-Garfinkle, S., Langdon, E.M., Taylor, N., Occhipinti, P., Bridges, A.A., Brangwynne, C.P., and Gladfelter, A.S. (2015). RNA controls PolyQ protein phase transitions. Mol Cell. *60*, 220–230.

STAR★METHODS

KEY RESOURCES TABLE

REAGENT or RESOURCE	SOURCE	IDENTIFIER
Antibodies		
GFP-Booster Alexa Fluor 488	Chromotek	Cat# gb2AF488-10
Bacterial and virus strains		
Sf9 cells	Expression systems	Cat#94-001F
Subcloning Efficiency DH5α Competent Cells	Invitrogen	Cat#18265017
Chemicals, peptides, and recombinant proteins		
Cytochalasin-D	Sigma-Aldrich	Cat# C8273
1,6 Hexanediol (1,6-HD)	Sigma-Aldrich	Cat# 240117
RNase A	Roche	Cat# RNASEA-RO
cOmplete Protease Inhibitor Cocktail, EDTA-free	Roche	Cat#5056489001
1X Dulbecco's Phosphate Buffered Saline (PBS) solution without $MgCl_2$	Sigma-Aldrich	Cat# D8537
Potassium chloride	Merck	Cat#104935
DTT	Fermentas Life Sciences	Cat#R0862
Benzonase	Produced in-house	N/A
Tris	Carl Roth	Cat# 5429
Amylose resin	NEB	Cat# E8021S
EDTA	Roche	Cat# 105063
Pipes	Applichem	Cat# A1079
monoGFP	Produced in-house	N/A
Activation Buffer (AB)	York-Andersen et al., 2015	N/A
Schinder's *Drosophila* medium	Gibco	Cat# 21720024
'Wash Buffer A' for Stellaris RNA FISH	LG Biosearch Technologies	Cat# SMF-WA1-60
'Hybridisation Buffer' for Stellaris RNA FISH	LG Biosearch Technologies	Cat# SMF-HB1-10
'Probe Hybridisation Buffer' for HCR V3.0	Molecular Instruments	Bundled with custom probe set
'Probe Wash Buffer' for HCR V3.0	Molecular Instruments	Bundled with custom probe set
'Amplification Buffer' for HCR V3.0	Molecular Instruments	Bundled with custom probe set
SlowFade Diamond Antifade Mountant with DAPI	Thermofisher Scientific	S36934
Protease inhibitor cocktail	Roche	Cat# CO-RO
Amylose Resin	New England Biolabs	Cat# E8021S
PreScission Protease GST 3C	GE Life Sciences	Cat# GE27-0843-01
Polyethene Glycol – 2000	Merck	Cat#817018
Recombinant GFP-Me31B protein	This paper	N/A
Recombinant GFP-Me31BΔN-ΔC protein	This paper	N/A
Critical commercial assays		
Size exclusion chromatography using a HiLoad 16/600 Superdex 200 pg	GE Life Sciences	Cat# GE28-9893-35
Amicon Ultra-0.5 Centrifugal Filter Unit	Millipore	Cat# UFC5030
Deposited data		
Raw set of disordered regions from the *Drosophila* proteome	This paper	https://github.com/holehouse-lab/supportingdata/tree/master/2021/sankaranarayanan_me31b_2021

(*Continued on next page*)

Continued

REAGENT or RESOURCE	SOURCE	IDENTIFIER
**Experimental models: Organisms/strains**		
D. melanogaster: y[1] w[*]; P{w[+mC]= PTT-GB}me31B[CB05282] (Me31B::GFP)	Bloomington Drosophila Stock Centre (Buszczak et al., 2007)	BDSC: 51530 FlyBase: FBst0051530
D. melanogaster: y,w, bcd-(ms2)$_6$ (18), bcd-(ms2)$_6$ (4); hsp83-MCP-RFP(4a)	Weil et al., 2006	N/A
D. melanogaster: GFP::Tral	Drosophila Genomics Resource Centre (Morin et al., 2001)	DGRC: 110584 Flytrap:G00089;DGRC:110584; RRID:DGGR_110584
D. melanogaster: y[1]; P{y[+mDint2] w[BR.E.BR]= SUPor-P} tral[KG08052] ry[506] / TM3, Sb[1] Ser[1] (tral^1)	Bloomington Drosophila Stock Centre (Wilhelm et al., 2005)	BDSC: 14933FlyBase: FBgn0041775
D. melanogaster: w[1118]; Df(3L)ED4483, P{w[+mW.Scer\FRT.hs3]=3'.RS5+3.3'} ED4483/ TM6C, cu[1] Sb[1]	Bloomington Drosophila Stock Centre (Wilhelm et al., 2005)	BDSC: 8070FlyBase: FBab0035731
**Oligonucleotides**		
Custom Stellaris FISH Probes for the 3'UTR of *bcd* RNA	Stellaris	See Table S2 (supplemental information).
Molecular Instruments Custom HCR Probe for *hb* RNA	Molecular Instruments	DNA Custom kit Drosophila melanogasterlot number: 2690/B795GenBank: NM_169234.2, Alexa647, v3.0 kits
**Recombinant DNA**		
Recombinant GFP-Me31B plasmid	This paper	N/A
Recombinant GFP-Me31BΔN-ΔC plasmid	This paper	N/A
**Software and algorithms**		
ImageJ	Schneider et al., 2012	https://imagej.nih.gov/ij/
ImageJ Plugin Trackmate	Tinevez et al., 2017	https://imagej.net/plugins/trackmate/
Rstudio/ R software	RStudio Team 2021	http://www.rstudio.com/.
HullRad	Fleming and Fleming, 2018	N/A
ABSINTH implicit solvent model	Vitalis and Pappu, 2009	N/A
CAMPARI Monte Carlo simulation (v3.0)	http://campari.sourceforge.net/V3/index.html	N/A
SWISS-MODEL	Waterhouse et al., 2018	N/A
SOURSOP	https://soursop.readthedocs.io/	N/A
MDTraj	McGibbon et al., 2015	N/A
DSSP Algorithm	Kabsch and Sander, 1983	N/A
Protfasta	https://protfasta.readthedocs.io/	N/A
Metapredict	Emenecker et al., 2021	N/A
LocalCIDER	Holehouse et al., 2017	N/A
PIMMS simulation engine	Martin et al., 2020	N/A
**Other**		
Iberian recipe fly food	Produced in-house	N/A
Oil 10 S (95 Series Halocarbon Oil)	VWR Chemicals	N/A
Olympus FV3000 Confocal Laser Scanning Microscope	Olympus	FV3000
DeltaVision Core widefield microscope	Applied Precision, LLC	DeltaVision Core
Custom built dual-trap optical tweezer instrument	Jahnel et al., 2011	N/A

RESOURCE AVAILABILITY

Lead contact

Further information and requests for resources and reagents should be directed to and will be fulfilled by the lead contact Timothy T. Weil (tw419@cam.ac.uk)

Materials availability

Reagents generated in this study are available upon request.

Data and code availability

- All other data reported in this paper will be shared by the lead contact upon request.
- All original code has been deposited at [https://github.com/holehouse-lab/supportingdata/tree/master/2021/sankaranarayanan_me31b_2021] and is publicly available as of the date of publication.
- Any additional information required to reanalyze the data reported in this paper is available from the lead contact upon request.

EXPERIMENTAL MODEL AND SUBJECT DETAILS

Drosophila stocks

The following transgenic lines were used in this paper:

Me31B::GFP (BDSC 51530, (Buszczak et al., 2007)), *h*sp83-MCP-RFP and *bcd*-(ms2)$_6$ (Weil et al., 2006), GFP::Tral (DGRC 110584, (Morin et al., 2001)), *tral*[1] (BDSC 14933) and *Df(3L)ED4483* (BDSC 8070) (Wilhelm et al., 2005).

Fly stocks were maintained at 25°C on Iberian recipe fly food as per standard procedure. Randomly selected, healthy, adult female flies typically 2-3 days after eclosing, with the required genotype for each experiment, that had not been subjected to previous experimental procedures were fed on yeast for two days at 25°C prior to dissection of ovaries or collection of embryos. For fattening, approximately 20 females and 10 males were put in a vial together. For embryo collection, 50-100 females and 20-40 males were placed in a cage together.

METHOD DETAILS

Oocyte sample preparation

Mature oocytes from fattened female flies were dissected (Weil et al., 2012b; Derrick et al., 2016) into 10S oil (95 halocarbon) on a 22 mm by 40 mm cover slip for live imaging. For extrusion assays, membranes of dissected mature oocytes were poked and ruptured using sharp forceps to extrude the oocyte contents into the oil. Extruded material was then subjected to live imaging.

Live imaging

Live imaging of *in vivo* and *ex vivo* P bodies, including all Fluorescent Recovery After Photobleaching experiments, were performed on the Olympus FV3000 microscope using the 1.35 NA, 60X silicone objective at a room temperature of 20°C (Note: subtle changes in temperature can affect P body recovery kinetics). For all *in vivo* experiments, P bodies in the anterior to mid-lateral region of the mature oocyte were imaged. Live imaging of recombinant Me31B condensates, induced on 35mm glass bottom MatTek dishes, was performed on the DeltaVision Core widefield microscope using a 1.4 NA, 60X oil immersion objective.

Pharmacological treatments

Mature oocytes mounted in oil on a 22 mm by 40 mm coverslip and set up under the microscope were treated with one or two drops of 10 μg/ml cytochalasin-D (Sigma-Aldrich) or 5% 1,6-HD (Sigma-Aldrich) or 500 ng/ml RNase A (Sigma-Aldrich) mixed in 1X Dulbecco's Phosphate-buffered saline (PBS) solution without $MgCl_2$ (Sigma-Aldrich), or home-made activation buffer (3.3 mM NaH_2PO_4, 16.6 mM KH_2PO_4, 10 mM NaCl, 50 mM KCl, 5% PEG 8000, 2 mM $CaCl_2$, pH 6.4; York-Andersen et al. 2015) using a glass pipette. Me31B or Tral labeled P bodies before and after treatment were then imaged. For salt experiments, mature oocytes were extruded into various concentrations of $MgCl_2$ or NaCl mixed in 1X PBS for 15 minutes before being subjected to live imaging. In the case of excessive movement of the oocytes or extruded material during addition of solutions, the focal plane of interest was adjusted accordingly, and imaging was performed.

Protein purification

The plasmid backbones for the production viruses and the SF9 insect cells for the purification of the recombinant proteins were provided by the protein purification facility at the MPI-CBG in Dresden. The recombinant Me31B, (wild-type (WT) and Me31BΔN-ΔC (mutant)), were cloned using established cloning techniques, tagged with a monomeric GFP (produced in-house), expressed in and purified from SF9 insect cells using the FlexiBAC baculovirus vector system (Lemaitre et al., 2019). Cell lysis was performed using a LM20 microfluidizer in lysis buffer containing 50 mM Tris/HCl pH 7.6, 2 mM EDTA, 1x EDTA-containing protease inhibitor cocktail (Roche), 1 M KCl, 5% glycerol, 3 μg/L benzonase (degrades nucleic acids), 1 mM DTT. The soluble lysate fraction was collected after centrifugation for 1 hour at 16000 rpm (Beckman Coulter JA-25.50) at 4°C. MBP-tagged protein was captured by gravity flow affinity chromatography using amylose resin (New England Biolabs). Captured protein was washed with wash buffer (50 mM Tris/HCl pH 7.6, 2 mM EDTA,1 M KCl, 5% glycerol, 1 mM DTT, 3 ug/L benzonase) and eluted using wash buffer containing 20 mM maltose. The eluted protein was incubated with GST 3C – PreScission protease (1:50) at room temperature for 2 hours to cleave off affinity tags. Samples were applied to size exclusion chromatography using a HiLoad 16/600 Superdex 200 pg (GE Life Sciences) on an Akta pure

chromatography system in 50 mM Tris/HCl pH 7.6, 2 mM EDTA,1 M KCl, 5% glycerol, 1 mM DTT. Proteins were finally concentrated using an Amicon Ultra centrifugal-500-30K filter at 4000 xg. Aliquots were flash frozen and stored at -80°C.

N terminal IDR sequence: MTEKLNSGHTNLTSKGIINDLQIAGNTSDDMGWKSKLKLPPKDNRFKTT

C-terminal IDR sequence: SVGDTCNNSDLNNSANEEGNVSK

In vitro condensation assay

Stored protein samples were thawed and spun at 5,000 rpm for a minute to remove any residual precipitates. To induce Me31B condensates (WT and mutant), 7.5 μM recombinant GFP-Me31B protein was added to an Eppendorf tube containing the condensation buffer (50 mM KCl, 20 mM PIPES, pH 7, 1% PEG-2K). Note: Gentle tapping of the tube induced spherical condensates. Mixing the content with a pipette tip was avoided as it prevented droplet formation.

Optical tweezer experiments

Condensate fusions for wildtype or mutant condensates were quantified using a custom built dual-trap optical tweezer instrument (Jahnel et al., 2011). Condensates were induced in the condensation buffer containing 5% PEG-2K at 20 μM Me31B protein concentration for both WT and mutant condensates. Post condensation, two condensates were trapped using separate optical traps and brought into close contact to induce fusion.

Fluorescence recovery after photobleaching

For whole FRAP, Me31B/Tral labeled P bodies or *in vitro* Me31B condensates were entirely photobleached for 5 seconds using 40% laser intensity from the 405 nm laser channel. For internal FRAP, a small region within Me31B labeled *in vivo* P bodies or *in vitro* Me31B condensates was photobleached for 5 seconds using 40% laser intensity from the 405 nm laser channel. Time lapse series of Me31B fluorescence recovery was recorded every 30 seconds (*in vivo* P bodies) or 10 seconds (*in vitro* Me31B condensates) using the pre-bleach imaging parameters (minimal laser intensity using the 488 nm laser channel, 2 Airy unit pinhole, 20482048 pixels).

Single molecule fluorescence *in situ* hybridization

Fly preparation: Ovaries from fattened female flies were dissected into 5% 1,6-HD(Sigma-Aldrich) dissolved in 1x PBS and teased apart to allow for permeation of the oocytes by 5% 1,6-HD. Oocytes were then incubated for 30 minutes before being transferred into and incubated in Schneider's *Drosophila* medium (Gibco) for 1 hour, oocytes were then fixed as below.

Fixation: Ovaries from fattened female flies were dissected into Schneider's *Drosophila* medium and teased apart before being fixed in 1 ml of 4% paraformaldehyde for 15 minutes at room temperature. Oocytes were washed thoroughly with 0.2% PBST before hybridization. Embryos collected for 1.5 hours were dechorionated with 50% household bleach. The embryos were then washed thoroughly and were fixed in a solution containing 500 μl of 4% paraformaldehyde and 500 μl heptane at room temperature for 15 minutes, the paraformaldehyde was replaced with 100% methanol, and this was shaken vigorously to pop the vitelline membrane. All liquid was removed, and the embryos were rinsed in methanol, before being washed in 0.2% PBST.

Stellaris RNA fluorescence *in situ* hybridization: Custom Stellaris FISH Probes were designed against the 3' UTR of *bcd* mRNA (GenBank: NM_057477, GenBank: NM_169157, GenBank: NM_169159, GenBank: NM_176411, GenBank: NM_176410) by utilizing the Stellaris RNA FISH Probe Designer (Biosearch Technologies, Inc., Petaluma, CA) available online at www.biosearchtech.com/stellarisdesigner. Drosophila oocytes and embryos were hybridized with the bcd mRNA Stellaris RNA FISH Probe set labeled with Quasar 570 (Biosearch Technologies, Inc.), following the manufacturer's instructions for 'Drosophila embryos' available online at www.biosearchtech.com/stellarisprotocols.

Protocol adapted from Trovisco et al. (2016), briefly, fixed oocytes and embryos were washed using 'Wash Buffer A' before being hybridized with 500 nM Quasar 570 -conjugated antisense Stellaris probes for bcd RNA in hybridization buffer at 37 degrees overnight. The oocytes and embryos were then re-washed in 'Wash Buffer A' before being washed with 0.2% PBST. The sample was then incubated in GFP-Booster Alexa Fluor 488 (1:500 (Chromotek)) at room temperature for 1 hour in 1% PBST. Embryos were washed well with 0.2% PBST before being mounted in SlowFade Diamond Antifade Mountant with DAPI (Thermofisher Scientific).

Custom Stellaris FISH Probes for the 3'UTR of *bcd* RNA, adapted from Trovisco et al. (2016), see Table S2.

In situ hybridization chain reaction V3.0

Protocol adapted from molecular instruments (Choi et al., 2018). Drosophila embryos were hybridized with 2 μl of odd and even HCR probes for *hb* mRNA in 100 μl 30% hybridization buffer at 37 degrees overnight. The sample was then washed with 30% probe wash buffer at 37 degrees and SSCT at room temperature. 2 μl of B4 Hairpin 1 and Hairpin 2 conjugated to an Alexa 647 were snap-cooled by heating to 90 degrees and cooled in the dark before being added to 100 μl of amplification buffer, this was added to the Drosophila embryos and incubated in the dark for 2 hours. The sample was then washed with SSCT at room temperature followed by 0.2% PBST before the addition of GFP-booster 488 (1:500 (Chromotek)) at room temperature for 1 hour in 1% PBST. Embryos were washed well with 0.2% PBST before being mounted in Slowfade Diamond with DAPI (Thermofisher Scientific).

QUANTIFICATION AND STATISTICAL ANALYSIS

Optical tweezer experiments

For quantifying the scaled fusion time for WT condensates, firstly, a relaxation time constant was derived from the fusion process over time. The scaled fusion time was then calculated by dividing the relaxation time constant by condensate radii to express the fusion time as a function independent of condensate size. For mutant condensates, due to their rapid aggregation post condensation, fusion was not quantifiable.

Fluorescence recovery after photobleaching

Mean fluorescence intensities were estimated using the Fiji ImageJ software. For whole FRAP analysis, background correction was performed by dividing Me31B fluorescent intensities of bleached condensates by fluorescent intensities of unbleached, cytoplasm. For internal FRAP, background correction was performed by dividing Me31B fluorescent intensities of bleached region within condensates by fluorescent intensities of whole condensates.

For all FRAP series, statistical analysis, curve fitting and plotting was performed using Rstudio/R software. Data for each condition was averaged and standard deviation was calculated where applicable. Recovery fitting of the normalized mean intensity as function of time was fitted by the least square analysis to determine fit to the single exponential equation: Normalized intensity = $P \times (1 - e(-t/\tau)) + y_0$ where y_0 is the recovery plateau, t is time, τ is the time constant and P is the amplitude of the fluorescence change. To infer the spatiotemporal pattern of internal Me31B fluorescence recovery, kymographs were generated using the ImageJ plugin 'reslice' by measuring fluorescence across of a region of interest over time.

All statistical analysis was completed in R/R Studio, the distribution of all data sets were analyzed in R studio before statistical analysis to assure the data met the assumptions for the appropriate statistical test. Statistical analysis of the difference in recovery kinetics P bodies between the three stages of oogenesis (stage 7, stage 12 and stage 14) used a Students t-test (Figure S2).

Fluorescence intensity measurements

Analysis of Me31B::GFP and *bcd-RFP* fluorescence before and after PBS and 1,6-HD treatment was performed using ImageJ processing software. Identical imaging parameters were utilized during imaging and measurement of fluorescence using 'analyze particles' and 'measure' feature on ImageJ. Individual Me31B and *bcd* particles were manually counted and analyzed before and after treatment with 1,6-HD. Random particles (*bcd* and Me31B together) were analyzed at different time points to avoid any bias.

Aspect ratio, circularity and particle area

P body aspect ratio, circularity, and area were measured using the 'analyze particles' and 'measure' features in ImageJ. Aspect ratio values are measured as the ratio of the major axis of a particle to the minor axis of a particle which gives an estimate of particle morphology. Circularity refers to the "roundedness" of a particle (and is calculated using the formula - circularity = 4pi (area/perimeter^2) which gives an estimate of particle shape.

All statistical analysis was completed in R/R Studio, the distribution of all data sets were analyzed in R studio before statistical analysis to assure the data met the assumptions for the appropriate statistical test. Statistical analysis of the difference in area and circularity of P bodies between the oocyte and embryo used a Two-Sampled Wilcoxon (Mann-Whitney) test. Statistical analysis of the difference in circularity of P bodies in the oocyte before and after treatment with 1,6-HD used a Wilcoxon signed-rank test.

Apparent viscosity estimation

Protocol was adapted from: (Hubstenberger et al., 2013). P body viscosity was estimated from internal FRAP recovery kinetics. Apparent diffusion of $\sim$ 0.00071 $\mu m^2/s$ was estimated from the calculated half-maximum (62.79s) using the equation: $D \approx 0.224\omega^2/\ t^{1/2}$, where ω is the radius of the bleach region, t is the time. Using the equation η=(KbT)6πRhD, where Rh is the hydrodynamic radius (An approximate hydrodynamic radius of Me31B was estimated based on our all-atom simulations, with the hydrodynamic radius calculated using HullRad (Fleming and Fleming, 2018), and T is the temperature at which experiments were conducted (21°C), the apparent viscosity was estimated.

Particle displacement analysis

P bodies were tracked and their displacement calculated before and after the addition of Activation Buffer (AB) or Cytochalasin D. Prior to tracking the images were pre-processed with a rolling ball background subtraction. Individual P bodies were then tracked using the FIJI plugin Trackmate (Tinevez et al., 2017). A simple linear tracker was used to determine particle tracks, and statistical analysis of the track displacement was completed in R/R Studio, using a Wilcoxon Signed Rank Test. The distribution of the data was analyzed in R studio before statistical analysis to assure the data met the assumptions for the statistical test.

All-atom simulations

All-atom simulations were run with the ABSINTH implicit solvent model and the CAMPARI Monte Carlo simulation (V3.0)

(http://campari.sourceforge.net/V3/index.html) and with the ion parameters from previously published work (Mao and Pappu, 2012; Vitalis and Pappu, 2009). Preferential sampling is used such that the backbone dihedral angles of folded domains are held fixed, while all sidechain dihedral angles, and the backbone dihedrals of folded proteins are fully sampled. In this way, we *a priori*

ensure that the folded domains remain folded. While the combination of ABSINTH and CAMPARI is well-established route to obtain reliable ensembles of disordered regions, more positional restraints on folded domains have been used previously applied to obtain good agreement with experiment (Cubuk et al., 2021; Martin and Mittag, 2018; Martin et al., 2020; Newcombe et al., 2018).

Starting structures were generated first by constructing homology models of Me31B based on the DDX6 structure (PDB: 4CT5) using SWISS-MODEL (Waterhouse et al., 2018). N- and C-terminal IDRs were constructed using CAMPARI. For all simulations, disordered regions were started from randomly generated non-overlapping random-coil conformations, with each replica using a unique starting structure. Monte Carlo simulations evolve the system via a series of moves that perturb backbone and sidechain dihedral angles along with the rigid-body coordinates of both polypeptides and explicit ions. Simulation analysis was performed using SOURSOP (https://protfasta.readthedocs.io/) and MDTraj (McGibbon et al., 2015). The protein secondary structure was assessed using the DSSP algorithm (Kabsch and Sander, 1983).

Contact score analysis was performed by assessing the fraction of simulations in which two residues were in direct contact, a distance calibrated as 5.0 Å or shorter between heavy atoms. This fraction was divided by the analogous fraction computed from simulations in which all attractive molecular interactions (solvation effects, electrostatics, attractive component of the Lennard-Jones potential) were set to 0.0, in the so-called excluded volume (EV) limit (Holehouse et al., 2015).

All simulations were run at 10 mM NaCl, and PEG 310 K. Fifty independent simulations were run for a total of 80 million Monte Carlo steps with 5 million steps for equilibration. The system state saved every 100,000 steps. Each simulation generated 750 frames, generating a final ensemble of 37,500 frames. Where included, error bars are standard error of the mean over the fifty independent simulations.

Bioinformatics

Disordered regions were calculated using both Mobidb-lite (Necci et al., 2017) and with metapredict (Emenecker et al., 2021; Piovesan et al., 2021). Disordered regions were identified using consensus scores from Mobidib-lite with a minimum IDR length of 25 residues and 3 or more predictors predicting a region to be disordered. The raw set of disordered regions from the Drosophila proteome, along with analogous data for P body proteins is provided in the supplementary repository: https://github.com/holehouse-lab/supportingdata/tree/master/2021/sankaranarayanan_me31b_2021.

Sequence analysis was performed using localCIDER (Holehouse et al., 2017).

Coarse-grained simulations

Coarse-grained simulations were performed with the PIMMS simulation engine (Martin et al., 2020)https://paperpile.com/c/pRQFLB/sMrm+DzJc. Lattice-based Monte Carlo simulations afford a computationally tractable approach to sample systems with coexisting liquid phases, as has been applied in several different contexts (Boeynaems et al., 2019; Fei et al., 2017; Feric et al., 2016; Martin et al., 2020). Monte Carlo moves include chain translate, rotate, and local/global pivot moves.

Simulations were run using a simple representation scheme in which Me31B was represented as a five-bead model made up of two N-terminal beads, a single central bead, and two C-terminal beads (Figure S4G-A). In this way, the protein consists of intrinsically disordered region (IDR) beads and ordered domain (OD) beads. RNA is represented as a 20-bead homopolymer (Figure S4G-A). We emphasize that these models are designed to describe a class of phenomenon, as opposed to capturing features specific to Me31B over RNA binding proteins. Our simplification of RNA and protein not-withstanding, these simple models allow us to interrogate general behavior.

The strength of interactions between the three bead types is shown in Figures S4G-A and S4G-B. Units are in per kT (where k=1). The core key files and parameter files used to run these simulations are provided at https://github.com/holehouse-lab/supportingdata/tree/master/2021/sankaranarayanan_me31b_2021.

Protein:RNA and RNA:RNA interaction strengths are held fixed across all simulations, while the protein:protein interaction strength is systematically altered across the simulations shown in Figures S4G-A and S4G-B. The specific interaction strengths were chosen to qualitatively reflect insights from experimental work - i.e. OD:OD interaction is stronger than OD:IDR interaction, with IDR:IDR interaction being the weakest. We also assume both OD and IDR beads can interact with RNA, and that RNA:RNA interaction is repulsive. As a final note, we anticipate that RNA:RNA interactions plays an additional role in P body stability, assembly, and disassembly. However, for our initial model, absent of other specific information, we avoided adding more tunable parameters to develop a simple yet physically reasonable.

All simulations were run with 800 Me31B protein molecules. Simulations with RNA were also run with 50 RNA molecules. These numbers were chosen to ensure that reasonable statistics on droplet recruitment could be obtained with a sufficiently large system where bona fide condensation occurs. Simulations were run on a 60 x 60 x 60 lattice with periodic boundary conditions, and simulation analysis was performed on the terminal 20% of the frames. Simulations were run for around 2.5 billion Monte Carlo moves, and three independent replicas were performed, such that error bars are the standard error of the mean on these replicas.

ADDITIONAL RESOURCES

No additional resources provided

Developmental Cell, Volume *56*

Supplemental information

Adaptable P body physical states differentially regulate *bicoid* mRNA storage during early *Drosophila* development

M. Sankaranarayanan, Ryan J. Emenecker, Elise L. Wilby, Marcus Jahnel, Irmela R.E.A. Trussina, Matt Wayland, Simon Alberti, Alex S. Holehouse, and Timothy T. Weil

Supplemental Information

Adaptable P body physical states differentially regulate *bicoid* mRNA storage during early *Drosophila* development

M.Sankaranarayanan, Ryan J. Emenecker, Elise L. Wilby, Marcus Jahnel, Irmela R. E. A. Trussina, Matt Wayland, Simon Alberti, Alex S. Holehouse, Timothy T. Weil

Ex vivo condensate rupture

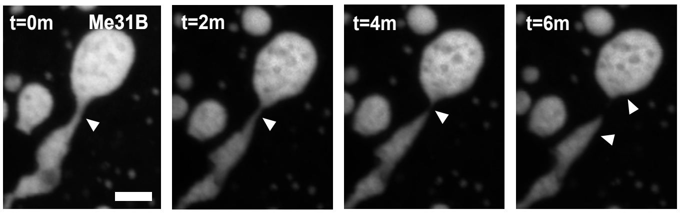

Figure S1. Extruded P bodies display condensate-rupture behavior. Related to Figure 2.

Extruded cytoplasm from a mature oocyte expressing Me31B::GFP. P bodies which have already fused are held together by a 'bridge' (t = 0 min, n = 10). Image sequence displaying 'pinching off' of the unstable bridge (white arrowheads point to the region of rupture). The two condensates resorb within a minute of bridge rupturing.

Scale bar: 2 µm.

A

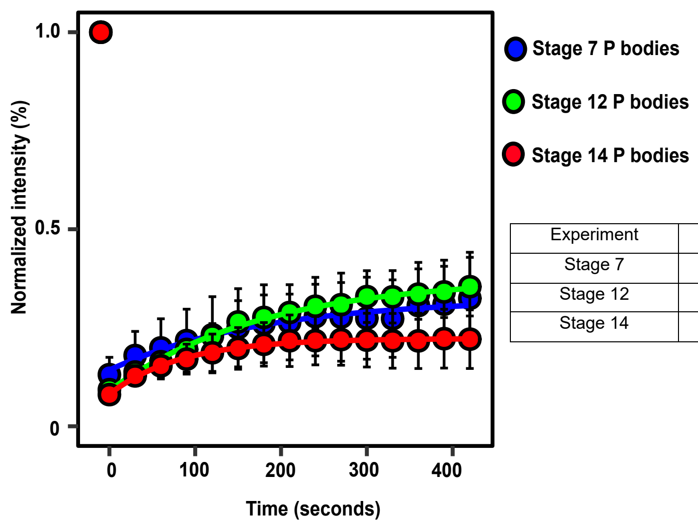

Experiment	Tau (seconds)	Mobile fraction	Immobile fraction
Stage 7	178.82	0.21	0.78
Stage 12	178.50	0.31	0.68
Stage 14	83.91	0.15	0.84

B

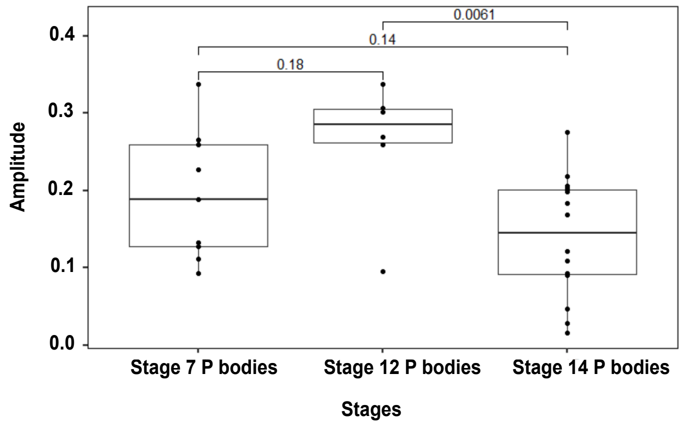

C

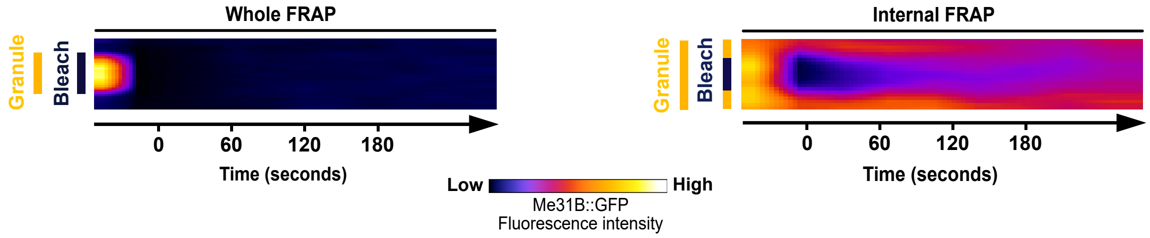

D

Experiment	Tau (seconds)	Mobile fraction	Immobile fraction
Whole FRAP	94.86	0.087	0.913
Internal FRAP	144.12	0.714	0.285

Figure S2. Me31B is mobile within P bodies but does not exchange with the cytoplasm. Related to Figure 2.

(A) Recovery profiles of whole FRAP of P bodies from stage 7,12, and 14 (mature oocyte) egg chambers expressing Me31B::GFP. Time constant (Tau), mobile, and immobile fractions of

Me31B estimated from recovery profiles of the whole FRAP (n = 9 for stage 7, n = 6 for stage 12, n = 16 for stage 14).

(B) Quantification of amplitude across P body recovery profiles from different stages of oogenesis reveals very little difference in the extent of recoveries FRAP (n = 9 for stage 7, n = 6 for stage 12, n = 16 for stage 14).

(C) Kymograph of *in vivo* P body from a mature oocyte expressing Me31B::GFP after whole FRAP shows no recovery, while kymograph of P bodies after internal FRAP displays recovery indicative of diffusion mediated recovery (whole FRAP n = 20, internal FRAP n = 24).

(D) Time constant (Tau), mobile, and immobile fractions of Me31B estimated from recovery profiles of whole FRAP or internal FRAP of P bodies from mature oocytes expressing Me31B::GFP (whole FRAP n = 20, internal FRAP n = 24).

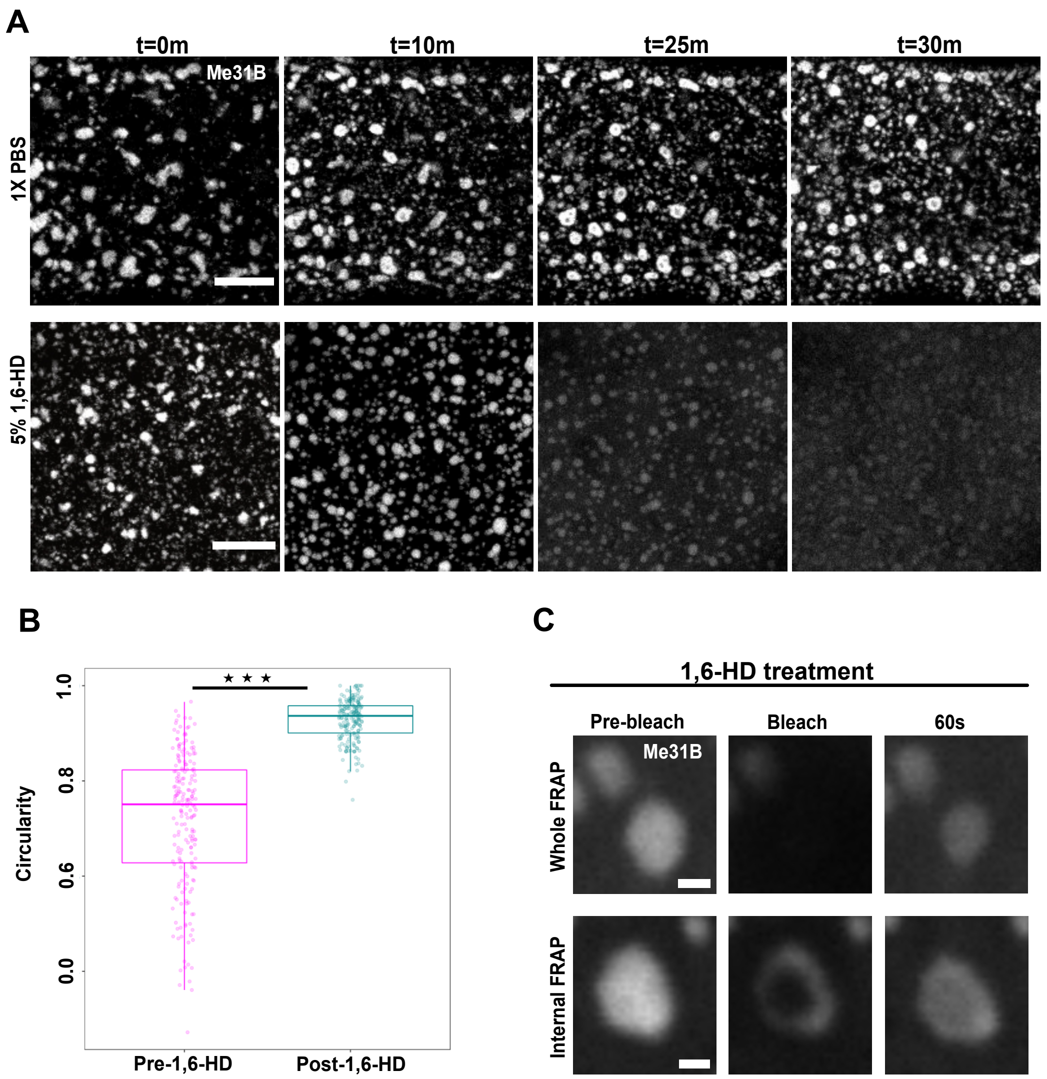

D

Experiment	Tau (seconds)	Mobile fraction	Immobile fraction
1,6-HD treated	10.16	0.641	0.358
Untreated	94.86	0.087	0.913

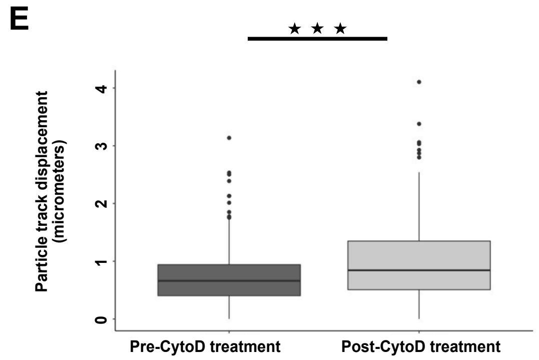

Figure S3. 1,6-HD and cytochalasin D treatments affect P body physical properties and dynamics. Related to Figure 3.

(A,C) Mature oocytes expressing Me31B::GFP.

(A) Time series of mature oocytes treated with 1X PBS (control) or 5% 1,6-HD shows that P bodies remain condensed in the control treatment while 1,6-HD leads to the dissolution of P bodies over time (n = 20 mature oocytes). Maximum projection 5 μm.

(B) Quantification of P body circularity prior to and after 1,6-HD treatment shows a significant increase in P bodies exhibiting spherical morphology post treatment (n = 200 P bodies, p<0.0001).

(C) Time series of P body condensates subjected to whole (n = 12) or internal (n = 3) FRAP after 1,6- HD treatment, both displaying rapid fluorescence recovery.

(D) Time constant (Tau), mobile, and immobile fractions of Me31B estimated from recovery profiles after being subjected to whole FRAP before or after treatment with 1,6-HD from mature oocytes expressing Me31B::GFP (n = 12).

(E) Quantification of particle displacement using TrackMate (Tinevez et al., 2017) software shows that P bodies in mature oocytes expressing Me31B::GFP after cytochalasin D treatment have increased spatial displacement compared to untreated P bodies (pre-treatment n = 674, post treatment n = 623 , p<0.0001).

Scale bar = 5 μm (A), 1 μm (C).

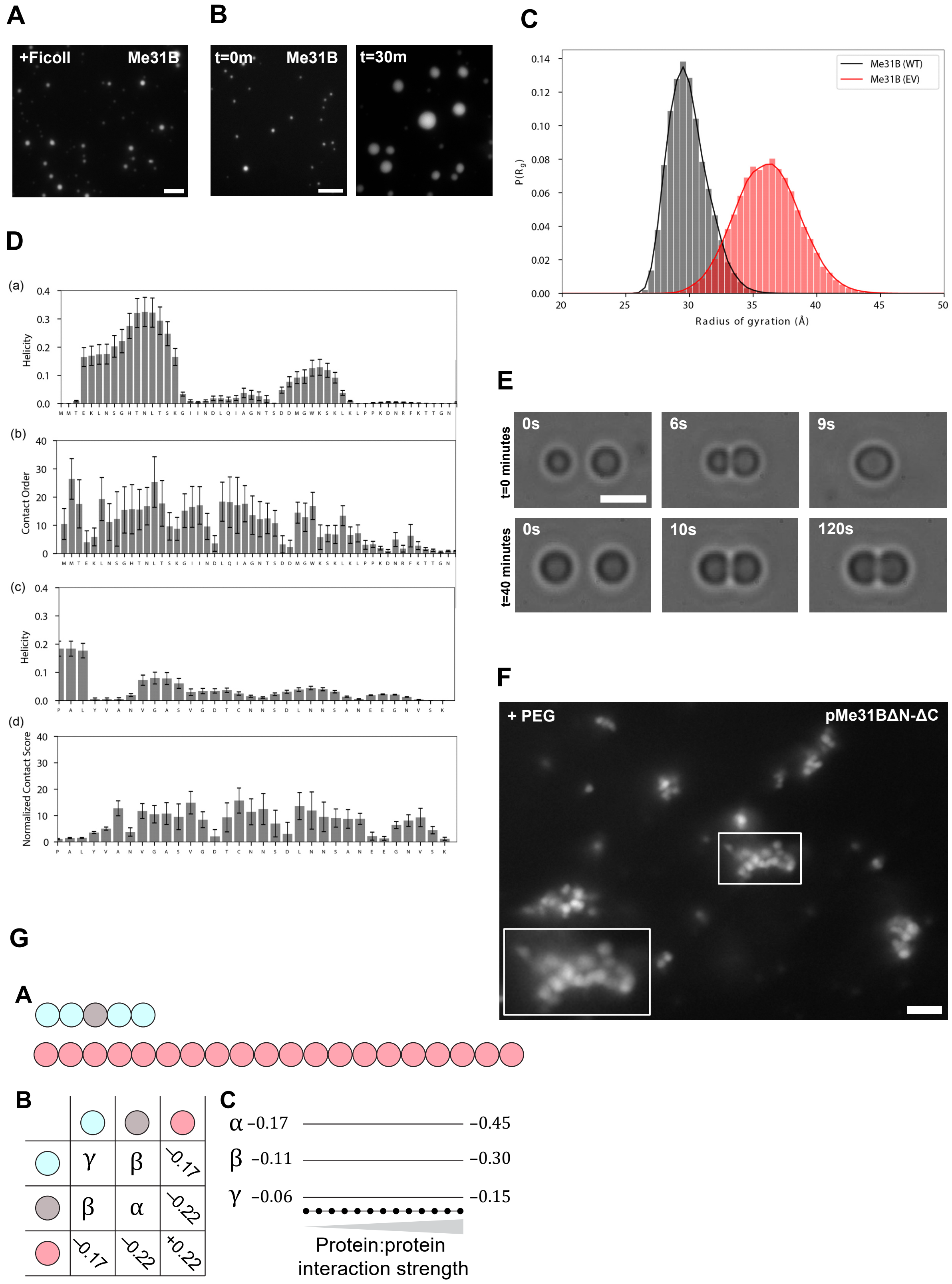

Figure S4. Interaction of IDRs with folded domains regulates Me31B physical state *in vitro*. Related to Figure 4 and Figure 6.

(A) 7.5 µm purified GFP-pMe31B in the presence of 1% Ficoll readily forms phase separated condensates (n = 10 independent replicates).

(B) Time series of purified GFP-pMe31B (7.5 μm) shows increase in size of condensates over 30 minutes (n = 10 independent replicates).

(C) Black bars show the Rg distribution for full-length Me31B under standard simulation conditions (full Hamiltonian (WT)), while red bars show the analogous distribution of the radius of gyration for simulations performed in which all attractive interactions are turned off (excluded volume (EV)). The full Hamiltonian simulations are substantially more compact, with an ensemble average radius of gyration of 30.1 Å, compared to 36.4 Å for the EV simulations. This compaction of the global dimensions originates from favorable interaction between the two IDRs and the folded domains.

(D) Local helicity (a,c) and intramolecular contacts (b,d) quantified on a per-residue basis for the NTD (a,b) and CTD (c,d). The NTD possess two short transient helices (4-17 and 31-37), while the CTD is entirely devoid of secondary structures. The NTD engages in more extensive intramolecular interactions than the CTD, as quantified by the normalized contact score (d) (see methods, larger values mean more contacts per residue). In both cases, the IDRs engage relatively uniformly, as opposed to via a specific motif. This implies broad and non-specific interactions between the IDRs and the folded domains.

(E) Time series of GFP-pMe31B condensate coalescence using optical traps. Rapid fusion of condensates is observed pre-gelation while condensates fail to fuse post-gelation (n = 25).

(F) pMe31B with both IDRs deleted (pMe31B ΔN-ΔC), in the presence of 1% PEG, fails to coalesce and rather forms amorphous aggregates (n = 10 independent replicates).

(G) Topology of Me31B (top) and RNA (bottom) molecules used in coarse-grained simulations (A)**.** Basic interaction table showing relative bead-bead interaction strengths with protein:protein bead interactions defined in terms of parameters (B). Protein:protein interaction parameters scale uniformly between max and min values (C).

Scale bar = 5 μm (A,B,F), 2 μm (E).

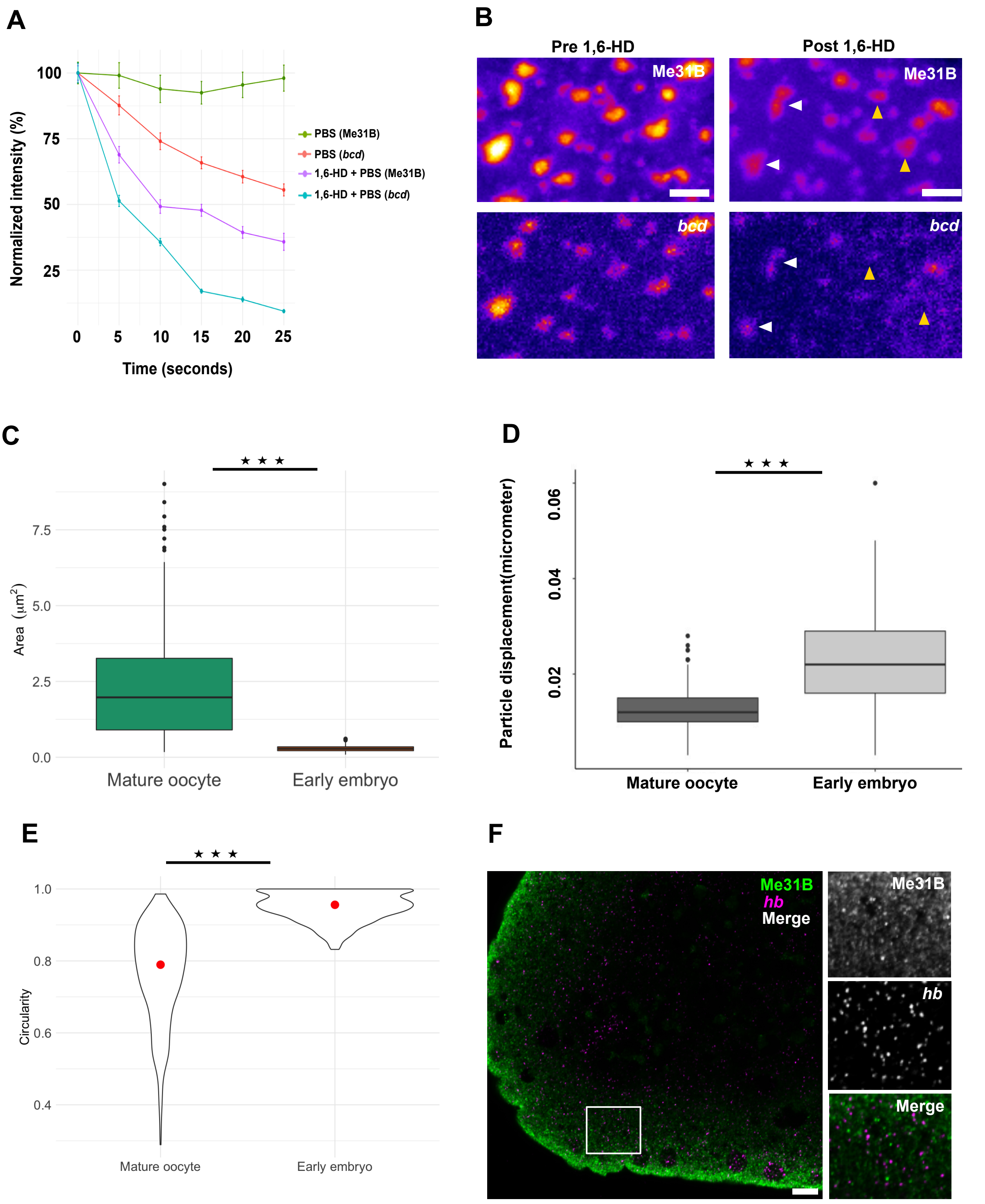

Figure S5. Egg activation modulates P body physical properties. Related to Figure 6.

(A) Quantification of images in Figure 6D and E. P body and *bcd* mRNA fluorescence in the presence of PBS (n = 35 Me31B and *bcd* particles) or 1,6-HD (n = 55 Me31B and *bcd* particles).

(B) Mature oocytes expressing Me31B::GFP, *hsp83-MCP-RFP,* and *bcd*-(*ms2*)$_6$ treated with 1,6-HD showing differential relationship of P bodies and *bcd* mRNA association (n = 55). In

some cases, *bcd mRNA* is retained in condensed P bodies post 1,6-HD addition (while arrowheads), while in others *bcd* is dispersed from condensed P bodies (yellow arrowhead).

(C) Quantification of P body sizes between mature oocytes and early embryos expressing Me31B reveals a significant decrease in embryonic P bodies (n = 400, p<0.0001).

(D) Quantification of particle displacement using TrackMate software shows that P bodies in the early embryo (n = 169) show increased track displacement compared to P bodies in the oocyte (n = 332, p<0.0001).

(E) Quantification of P body circularity from embryos expressing Me31B::GFP show that they are significantly more spherical compared to P bodies in the mature oocyte (n = 400, p<0.0001).

(F) Early embryo expressing Me31B::GFP labelled with GFP-Booster and FISH for *hb* mRNA shows no co-localization of P bodies and *hb*. Inset shows a zoomed in version of *hb* mRNA and P body distribution (n = 10 early embryos). Maximum projection 5 µm.

Scale bar = 2 µm (B), 5 µm (F).

Symbol	Name	Fly Base ID	UniProt ID
me31B	maternal expression at 31B	FBgn0004419	P23128
tral	trailer hitch	FBgn0041775	Q9VTZ0
bru1	bruno 1	FBgn0000114	O02374
cup	cup	FBgn0000392	Q9VMA3
DCP1	Decapping protein 1	FBgn0034921	Q9W1H5
DCP2	Decapping protein 2	FBgn0036534	Q5U127
edc3	Enhancer of decapping 3	FBgn0036735	Q9VVI2
eIF4E1	eukaryotic translation initiation factor 4E1	FBgn0015218	P48598
exu	exuperantia	FBgn0000615	P28750
lost	lost	FBgn0263594	Q9VN21
Patr-1	Protein associated with topo II related - 1	FBgn0266053	Q9VEN9
Hrb27C	Heterogeneous nuclear ribonucleoprotein at 27C	FBgn0004838	P48809
orb	oo18 RNA-binding protein	FBgn0004882	Q8IMZ2
pcm	pacman	FBgn0020261	Q9XZU2
stau	staufen	FBgn0003520	P25159
sqd	squid	FBgn0263396	Q08473
AGO3	Argonaute 3	FBgn0250816	Q7PLK0

Table S1. List of *Drosophila* P body proteins. Related to Figure 5A.

5'-GAAACTCTCTAACACGCCTC-3'
5'-ACAGTGGTTAACCTAAAGCT-3'
5'-TGGTATTTGTACAATCAGGA-3'
5'-CTTTCTACGCGTAGATATCT-3'
5'-ACGGATCTTAGGACTAGACC-3'
5'-AAACTTCCCTGGGAACCATT-3'
5'-CTGCTGACTAGGCTAGTACA-3'
5'-GATATGCACTGGAATCCGTG-3'
5'-GAGTTAACTGGAGTATCACT-3'
5'-AGCGTATTGCAGGGAAAGTA-3'
5'-CACCCAGATACATCTAAGGC-3'
5'-CATATTCCCGGGCTTTAGTG-3'
5'-TGGCCTCAAATGTAACTGGT-3'
5'-ACTTTCCATGGAATACGCTT-3'
5'-ATTTCCGAAATGTGGGACGA-3'
5'-AGAAGATTTTCTTGCTGGCT-3'
5'-GTACAGTTTTTAGCTATGTC-3'
5'-ATGAGATTACGCCCAAGAGA-3'
5'-ATGTTCGATCTTTAAGGGTA-3'
5'-ACACTTTGGCATAGCATAGA-3'
5'-GCGCAAATGTTTGATTATGT-3'
5'-TTGCTGACTATTCTTGGTCA-3'
5'-ACAAATGGTCTGCATTGATT-3'
5'-TGATAGTTATTCCGTTTGGC-3'
5'-ATGCTCTTCTTAGTGATGTA-3'
5'-ACTTGAGGCCTAACAGATTG-3'
5'-ACAACATCAAAGGTGCAGCA-3'
5'-ATTTACCCGAGTAGAGTAGT-3'

Table S2. Custom Stellaris® FISH Probes for the 3'UTR of *bcd* RNA. Related to Figure 6 and Figure 7 (adadpted from Trovisco et al., 2016).
